# Optimized processing of Gardenia Fruits with ginger juice: Unveiling therapeutic mechanisms for cholestatic liver injury through TLR4/NF-κB, FXR/PPAR-α, and PI3K/AKT/GSK-3β

**DOI:** 10.1371/journal.pone.0330189

**Published:** 2025-09-16

**Authors:** Chenzi Lyu, Xianglong Meng, Juan Wang, Haifeng Shao, Yang Wang, Yong-Ki Park, Shuosheng Zhang, Hyo Won Jung

**Affiliations:** 1 Department of Herbology, College of Korean Medicine, Dongguk University, Gyeongju, Republic of Korea; 2 College of Chinese Materia Medica and Food Engineering, Shanxi University of Chinese Medicine, Jinzhong, Shanxi, China; 3 Shanxi Key Laboratory of Traditional Herbal Medicines Processing, Jinzhong, Shanxi, China; 4 Korean Medicine R&D Center, Dongguk University, Gyeongju, Republic of Korea; Ann and Robert H Lurie Children's Hospital of Chicago / Northwestern University Feinberg School of Medicine, UNITED STATES OF AMERICA

## Abstract

The study aimed to optimize the processing conditions of Gardeniae Fructus with ginger juice (GFPG) and confirm its therapeutic effects and pharmacological mechanisms on cholestatic liver injury. Processing conditions were optimized using response surface methodology (RSM) and thermal analysis, focusing on geniposide content as a key active compound. Variables included processing time, moistening time, and the solid-liquid ratio. Optimal conditions were: ginger juice to Gardeniae Fructus ratio of 8:1 (w/v), processing temperature of 208 °C, moistening time of 3 hours, and processing time of 5 minutes. Pharmacological mechanisms were analyzed through network pharmacology, molecular docking, and experimental validation using alpha-naphthyl isothiocyanate (ANIT)-induced cholestatic liver injury in mice and lipopolysaccharide (LPS)-stimulated RAW264.7 cell models. *In vivo*, GFPG extract alleviated ANIT-induced cholestatic liver injury by improving liver function markers (AST, ALT, TBA, TBIL, DBIL) and modulating TLR4/NF-κB, FXR/PPAR-α, and PI3K/AKT/GSK-3β pathways. *In vitro*, it reduced LPS-induced production of inflammatory mediators (NO, TNF-α, IL-6, IL-1β) through TLR4/NF-κB pathway inhibition. This study established optimal processing methods for GFPG using RSM and thermal analysis, providing robust quantitative parameters. GFPG demonstrated significant therapeutic effects in cholestatic liver injury models, indicating its potential as a candidate for developing treatments for cholestatic hepatitis.

## Introduction

Herbs with medicinal properties have been widely utilized in the food and health industries for centuries, contributing to both nutrition and wellness. Recent advancements in herbal processing and extraction technologies focus on improving herb efficacy, optimizing active ingredient yield, and reducing side effects [1]. Thermal analysis is a technique employed in herbal processing to evaluate how the physical properties of herbs change with temperature under controlled settings [2]. Many substances undergo physical changes such as decomposition, dehydration, oxidation, reduction, and sublimation under certain temperature conditions during the heating process. The temperature (T) and percentage (%) of mass change of herbal substances during heating vary with the structure and composition of the material. Consequently, thermal analysis technology has found application in contemporary research on herbal processing within Traditional Chinese Medicine (TCM). It is utilized in thermogravimetry (TG) and derivative thermogravimetry (DTG) to simulate the heating processes involved in herb processing [3]. Thermal analysis combined with response surface methodology (RSM) is also used in herbal processing research for fire quantification using processing parameters.

The mature fruit of *Gardenia jasminoides* Ellis (Rubiaceae family; Gardeniae Fructus, GF) have been used in TCM as a medicinal and edible herb, known for their heat-clearing, inflammation, jaundice, edema, fever, and hepatic disorders [4]. In TCM theory, GF is classified as having a “cold” drug properties, which may restrict its use among certain populations [5]. In contrast, *Zingiber officinale* (ginger) is considered “warm” drug properties, with functions such as relieving the exterior, dispelling cold, and stopping nausea. By combining ginger and GF through a specialized processing technique, a novel product, “Gardeniae Fructus processed with ginger juice” (GFPG), is developed. This formulation retains the pharmacological effects of GF while mitigating its cold nature. Actually, historical records of the clinical application of GF processed with ginger juice can be traced back to the Song Dynasty in ancient China. In the medical text *Chan Bao Za Lu* (Miscellaneous Records of Treasure Production) [6], the use of ginger juice in the preparation of GF for therapeutic purposes was documented, highlighting its long-standing tradition in herbal application.

Cholestatic hepatitis can arise from multiple factors, including oxidative stress, mitochondrial dysfunction, inflammation, and cell apoptosis. This condition can progress to liver fibrosis and cirrhosis [7], leading to impaired bile secretion and excretion, ultimately resulting in bile acid accumulation in the liver. Unfortunately, no specific or safe drugs are currently available for cholestatic hepatitis. GF has been used in traditional clinics to improve the symptoms of cholestatic hepatitis via liver protection [8]. It is used in the prescription “Yin-Zhi-Huang”, which originates from the classic formula for treating cholestasis found in the ancient Chinese medical book “*Shanghan Lun*” [9]. Although there are some reports on the liver-protective effects of GF, the synergistic effects of GFPG on cholestatic liver disease remain scientifically unexplored.

In this study, we examined the pyrolysis characteristics of GFPG alongside its main active compounds during thermal processing, aiming to establish optimal processing conditions. We also identified molecular targets for the efficacy of GFPG against cholestatic hepatitis through network pharmacology and molecular docking analysis. Subsequently, we explored the therapeutic effects of GFPG on liver injury caused by cholestatic hepatitis in mice and examined its mechanisms related to inflammation in RAW264.7 cells.

## Materials and methods

### Materials

Professor Shuosheng Zhang from Shanxi University of Chinese Medicine verified the dried Gardeniae Fructus (GF) sourced from Beijing Tongrentang (Jinzhongchain store) in Shanxi, China, as Gardeniae Fructus herbal materials (Batch No. 170821007). Voucher specimens, identified by the numbers SXTCM-Lyu-2022006, are kept at the Herbarium of Shanxi College of Traditional Chinese Medicine in Taiyuan, China. UDCA tablets were purchased from DAEWOONG PHARMACEUTICAL Co., Ltd (Seoul, Korea). Aspartate aminotransferase (AST) assay slide (LOT 496307) and Alanine aminotransferase (ALT) assay slide (LOT 4335510) were acquired from FUJIFILM Life Sciences Korea Co., Ltd (Seoul, Korea). The Alkaline Phosphatase (ALP) assay kit (A059-2–2), Direct Bilirubin (DBIL) assay kit (C019-2–1), Total Bilirubin (TBA) assay kit (E003-2–1), and Total Bilirubin (TBIL) assay kit (C019-1–1) were obtained from Nanjing Jiancheng Bioengineering Institute in Nanjing, China. Tumor necrosis factor alpha (TNF-α) (EM0183) ELISA Kit, Interleukin-1 beta (IL-1β) (EM0109) ELISA Kit were purchased from FineTest Biotech Inc. (Wuhan, China). Antibodies against iNOS (Cat No. 13120S, Dilution1:1000), NF-κB (cat No. 4764S, Dilution1:1000), p-NF-κB (Cat No. 3033S, Dilution1:1000), TNF-α (Cat No. 11948S, Dilution1:1000), IL-6 (Cat No. 12912S, Dilution1:1000), IL-1β (Cat No. 12426S, Dilution1:1000), FXR (Cat No. 72105S, Dilution1:1000), AKT (Cat No.9272S, Dilution1:1000), p-AKT (Cat No. 9271S, Dilution1:1000), and PI3K (Cat No. 4292S, Dilution1:1000) were purchased from Cell Signaling Technology (Danvers, MA, USA). Antibodies against Bax (Cat. sc-493, Dilution1:500), Bcl-2 (Cat. sc-7382, Dilution1:500), caspase-3 (Cat. sc-56053, Dilution1:500) were purchased from Santa Cruz Biotechnology (Dallas, TX, USA). Antibodies against TLR4 (Cat. 48–2300, Dilution1:1000) was purchased from Thermo Fisher Scientific Inc. (Waltham, MA USA). Antibodies against CYP7A1 (Cat. BS-2399R, Dilution1:1000) was purchased from Bioss Inc. (Woburn, MA USA). Horseradish peroxidase-conjugated goat anti-rabbit immunoglobulin G (IgG) antibody (cat.no. BR1706515, Dilution1:2000), anti-mouse IgG antibody (Cat No. BR1706516, Dilution1:2000), and β-actin (cat no. A1978, Dilution1:2000) were supplied by Sigma-Aldrich (Burlington, MA, USA). Geniposide reference substance (MUST-18032401, 99.03%), ursolic acid reference substance (MUST-17070501, 98.07%), chlorogenic acid reference substance (MUST-22111711, 99.82%), quercetin reference substance (MUST-23112010, 99.34%), and 6-gingerol reference substance (MUST-23112212, 98.38%) were acquired from Chengdu Manster Biotechnology Co., Ltd., China. Additionally, forty male ICR mice with SPF status, each weighing 18–20 grams, were obtained from Hana Experimental Animals Co. (Busan, Korea).

### Sample preparation for thermal analysis

GF powder was prepared by weighing 100 g of GF, crushing, and passing through a 40-mesh sieve. Ginger juice was prepared using a traditional method, utilizing fresh ginger that was thoroughly washed and cut into small pieces. The chopped ginger was combined with an equal volume of deionized water (1:1, v/v) and blended at high speed for 2 minutes until a homogeneous mixture was obtained. Subsequently, the mixture was filtered through a fine mesh sieve or cheesecloth to remove the ginger residue, and the filtered ginger juice was collected. The resulting ginger juice can be stored at 4°C and should be used within 48 hours.

Extracted samples of total iridoids, organic acids, and flavonoids were prepared following the methods outlined in a previous report [3].

To prepare the GFPG test powder, the GF powder was mixed with freshly prepared ginger juice at a mass-to-volume ratio (w/v) of 1:1, meaning 1 g of GF powder was combined with 1 mL of ginger juice. The mixture was thoroughly blended to ensure complete absorption of the ginger juice by the GF powder. The resulting mixture was then dried in an oven at 60°C for 12 hours until fully dried and powder-like in texture. The dried material was ground and sieved to obtain a homogeneous powder, which was used as the GFPG test sample in this study.

### Thermogravimetry analysis

Thermal analysis was conducted using a NETZSCH STA449-F5 simultaneous thermal analyser under specific conditions: a carrier gas composition of simulated air with a nitrogen (N₂) to oxygen (O₂) ratio of 4:1, 60 mL/min flow rate, 5 °C/min heating rate, and a target temperature set at 580 °C. The experimental conditions were established based on the pyrolytic characteristic of traditional processing techniques, with the thermodynamic endpoint set at 580°C to simulate the complete process. This temperature threshold has been experimentally validated to ensure full carbonization and decomposition of typical herbal components, covering all stages of pyrolysis. The samples of GF powder (30 ± 5 mg), extracts of total iridoids, total flavonoids, total organic acids, GFPG test sample powder, geniposide, ursolic acid, and chlorogenic acid as reference substances were evenly dispersed in the crucible. The pyrolysis characteristics were also studied under the same conditions [10] with three replicates.

### Single-factor study for establishment of optimal processing condition for GFPG preparation

An ABL-series multifunctional frying machine (25 L) from Lanzhou Apollo Electronic Equipment Co., Ltd. was used to prepare the GFPG samples. Briefly, five GF powder samples (100 g each) were placed in a container and mixed with ginger juice at ratios of 10:1, 9:1, 8:1, 7:1, and 6:1 (w/v). The samples were thoroughly mixed and moistened for 3 hours before being placed in a frying machine. They were fried at 208 °C for 5 minutes, then removed and allowed to cool in air. The final GFPG products were subsequently processed in various solid-to-liquid (w/v) ratios. Five pieces of GF (100 g each) were then taken, placed in a container, and ginger juice was added at a material-to-liquid ratio of 8:1 (w/v). The samples were mixed well, moistened for 3 h, placed in the frying machine, fried for 3, 4, 5, 6, 7, or 8 min at 208 °C, taken out, and cooled in air. Five other GF pieces (100 g) were also placed in a beaker, and ginger juice (8:1 w/v) was added. The samples were mixed well, moistened for 1, 2, 3, 4, or 5 h, placed in the frying machine, and fried at 208 °C for 3 min. The samples were then removed and cooled in air to obtain GFPG samples at different moistening times. High-performance liquid chromatography (HPLC) was used to analyse the geniposide content in all GFPG samples. (S1 File).

### Response surface analysis for establishment of optimal processing condition for GFPG preparation

For the single-factor test, three factors, the solid-liquid ratio (*A*), processing time (*B*), and moistening time (*C*), were selected as response variables. An experiment was conducted using Design Expert 8.0.6 software according to Behnken’s central composite experimental design principle. As the result, the content (*Y*) of geniposide was taken as the response value, and the processing technology was optimised by response surface analysis to obtain the optimal processing conditions which were verified in actual operation.

### Data analysis of GF and ginger from network pharmacology

The chemical components of ‘Gardeniae Fructus’ (Zhizi in Chinese) and ‘Ginger’ (Shengjiang in Chinese) were extracted from the traditional chinese medicine systems pharmacology database and analysis platform (TCMSP) [11]. To identify potential active ingredients in Gardeniae Fructus and ginger, a minimum oral bioavailability of 30% and a drug-likeness score of at least 0.18 were used. The TCMSP database provided target links, which were annotated using gene abbreviations from the UniProt database. Relevant targets for cholestatic hepatitis were identified from the Gene Cards database [12], using the search terms “cholestatic”, “cholestatic hepatitis” and “cholestatic liver disease”. Duplicate entries were eliminated to finalize the target information for cholestatic hepatitis (C-H).

### Network analysis of active targets and pathways of Gardeniae Fructus and ginger in cholestatic hepatitis

After screening, the potential active targets of GF and ginger were intersected with the targets associated with cholestatic hepatitis using the online platform jvenn (http://www.bioinformatics.com.cn/) [13]. A network diagram showing the intersection of targets and active ingredients was created using Cytoscape 3.9.0. To create a protein-protein interaction (PPI) network, the intersected targets were uploaded into the STRING database [14], visualized using Cytoscape 3.9.0, and analysed for degree values. GO functional enrichment and KEGG pathway analyses of the intersecting targets were performed using the Metascape database [15]. The Microbiota platform (http://www.bioinformatics.com.cn/) was used to display the top eight enriched biological processes (BP), cellular components (CC), molecular functions (MF), and KEGG pathways [16].

### Molecular docking analysis

Three active compounds—geniposide, chlorogenic acid, and quercetin—were selected for having the highest number of associated targets and subjected to molecular docking analysis with key potential targets such as AKT, IL-β, IL-6, and TNF-α identified from the PPI network. The SDF files of these active ingredients from GF and ginger were acquired from the PubChem database to serve as ligand files. Using AutoDock Tool software (Version 1.5.6, http://vina.scripps.edu/), protein receptor files from the PDB database (http://www.rcsb.org/) were subjected to hydrogenation. AutoDock Vina was used for molecular docking analyses, and the outcomes were calculated. The Affinity (kcal/mol) value represents the binding energies between the ligand and receptor. Binding energies below −5 kJ/mol indicate strong binding activity, with lower values suggesting a more favorable docking effect. All results were visualized using PyMOL software (https://pymol.org/2/) [17].

### HPLC analysis of active components in GFPG and GF extracts

In the RSM, it was determined the best processing technology for GFPG preparation. Each sample (200 g) of GFPG and GF was mashed, added water (1 L), boiled for 40 min, and filtered. Each filtrate was boiling with water (1.6 L) for 40 min, again and then filtering twice. The final filtrates were concentrated under reduced pressure at a temperature of 60 °C, and lyophilised in a freeze-dryer (IlShin Lab Co., Yangju, Korea) at −80 °C with a pressure of 5 mTorr. The extract of GFPG (yield = 29.12%) and GF (yield = 34.13%) were stored at 4 °C until experiments.

HPLC analysis was used to determine the levels of geniposide, chlorogenic acid, and quercetin in the GF and GFPG extracts. Also, the content of 6-gingerol was measured in the GFPG extracts.

### The impact of GFPG extract on inflammation in LPS-stimulated RAW264.7 cells was assessed

The RAW264.7 mouse macrophage cell line (TIB-71, ATCC, USA) was cultured in DMEM containing 10% FBS and 1% penicillin-streptomycin at 37°C with 5% CO₂. The optimal treatment concentrations of GFPG extract were established through a cell viability assay (S1 Fig.). Cells (1 × 10⁵/mL) were briefly treated with 0.5 or 1 mg/mL GFPG extract or 10 μmol/L dexamethasone (DEX) for one hour, followed by 1 μg/mL LPS stimulation to induce inflammation. The culture supernatants were harvested and were measured NO levels using an NO assay kit (Cat No. DG-NO500, Seoul, South Korea) at 540 nm. The NO levels was calculated using the standard calculation of nitrite.

### Preparation of a liver cholestasis mice model

**Fig 1:**
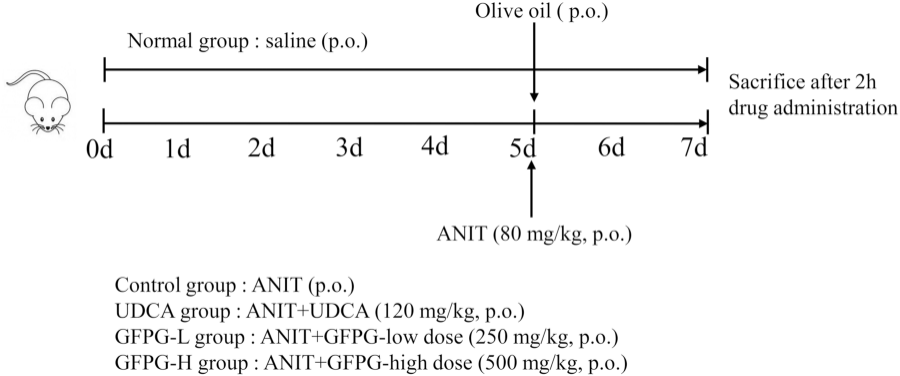
Establishing a mouse model for ANIT-induced liver injury related to cholestasis.

Forty male ICR mice were selected and acclimatized for 3 days, at a temperature of 22–26 °C, with a controlling humidity of 60–70%, and under light control with a lamp for 12 h/d. The animal experimental protocol for this study received approval from the Ethics Committee of Dongguk University (approval number IACUC-2024-06). Mice were randomly divided into five groups using a computer-generated method, with eight mice in each group. Group allocation was conducted by an independent researcher who was not involved in the subsequent experiments. The groups were as follows: normal, cholestasis-induced control (Control), UDCA-administered (UDCA, 120 mg/kg b.w.), GFPG extract low dose-administered (GFPG-L, 250 mg/kg b.w.), and GFPG extract high dose-administered (GFPG-H, 500 mg/kg b.w.). In this study, we used the body surface area (BSA) normalization method to convert the clinical doses of raw GFPG herbs (86–143 mg/kg) and UDCA (10 mg/kg) in adults to the treatment doses for mice. Notably, GFPG extract (yield is 29.12%.) was used in this study. Therefore, 500 mg/kg was set as the high dose, and a low-dose group (250 mg/kg) was added to investigate dose–response relationships and safety. Saline was administered orally to the normal and control groups, while the treatment groups received their specific treatments once daily for 7 days at a dose of 10 mL/kg. Body weight measurements were taken for all mice every two days. On the 5th day, one hour after drug administration, except for the normal group, all mice were treated with an ANIT solution (0.8 g ANIT in 100 mL olive oil, p.o.) at a dosage of 80 mg/kg of body weight to induce a cholestatic liver injury model [18]. The normal group was received olive oil only as a blank solvent (p.o.). Forty-eight hours’ post-induction, the mice were euthanized using inhalation of a gas mixture composed of 75% O_2_ and 25% N_2_O, administered with 5% isoflurane for more than 3 minutes. Death was confirmed by monitoring for signs such as the absence of chest movement, no palpable heartbeat, pale mucous membranes, lack of response to toe pinch, and changes in eye coloration (Fig 1). Whole blood samples were immediately collected via cardiac puncture after sacrifice, and the liver tissue was then obtained, weighed, and stored at −80°C for further study.

### Determination of the levels of serological biomarkers

Blood samples were centrifuged at 3000 rpm for 15 minutes to obtain the serum. Following the kit instructions, the serum samples from each group were analysed for serological biomarkers, including AST, ALT, ALP, direct bilirubin (DBIL), total bile acids (TBA), total bilirubin (TBIL), IL-1β, and TNF-α.

### Histopathological observation of liver tissue

Liver tissues were fixed with 4% paraformaldehyde, prepared paraffin-embedded blocks (Histocenter 3; SHANDON, UK), cut the sections using a rotary microtome, and mounted on microscope slides. The slides were stained with hematoxylin and eosin (H&E) and examined under a microscope for morphological differences (BX53, Olympus, Japan).

### Western blot analysis

Liver tissues or cells were homogenized in 100 μL of RIPA lysis buffer containing protease and phosphatase inhibitors. After vortexing for 1 min and incubating on ice for 10 min, the lysates were centrifuged at 12,000 rpm for 20 min to remove debris. Total protein concentrations were determined using a Bio-Rad protein assay kit (Bio-Rad Inc., CA, USA). Equal protein amounts (30 μg/sample) were separated by SDS-PAGE, transferred to nitrocellulose membranes, and blocked with 2% skim milk (Becton, Dickinson and Company, Franklin Lakes, NJ, USA). Membranes were incubated overnight at 4 °C with primary antibodies targeting iNOS, NF-κB, p-NF-κB, TNF-α, IL-1β, TLR4, IL-6, FXR, PPAR-α, CYP7A1, PI3K, AKT, p-AKT, GSK-3β, Caspase-3, Bax, Bcl-2, and β-actin. After three washes with TBS-T (1 × Tris-buffered saline containing 0.1% Tween® 20), membranes were incubated with secondary antibodies for 90 min, and protein bands were detected using the ChemiDoc MP Imaging System (Bio-Rad, Hercules, CA, USA). Band intensities were quantified with ImageJ software (Scion Corp., Frederick, MD, USA) and normalized to β-actin or total AKT and NF-κB levels for comparison.

### Statistical analysis

Thermal analysis data were visualized using Origin 8.0 (Origin Lab, Northampton, MA, USA), while *in vitro* and *in vivo* experimental results were analysed with GraphPad Prism (GraphPad Holdings, San Diego, CA, USA). Statistical comparisons between two groups were performed using the independent sample t-test and Tukey’s test, whereas variations among multiple groups were analysed via ANOVA. Non-parametric tests were used to analyse data that were not normally distributed, with a p-value of less than 0.05 considered statistically significant.

## Results

### Pyrolysis characteristics analysis

**Fig 2:**
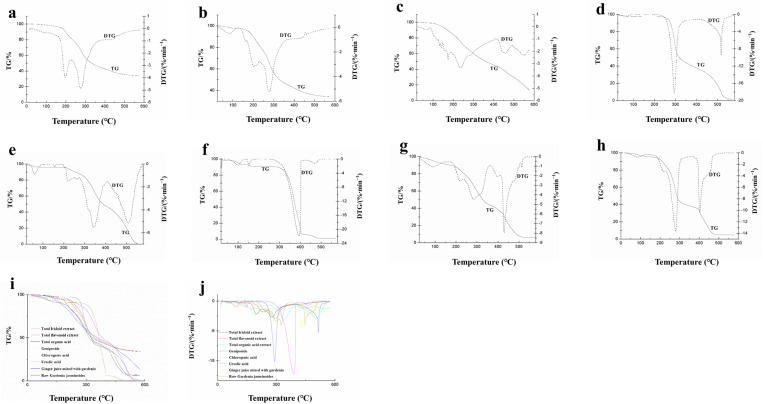
Pyrolysis characteristic curves of main compounds in GFPG. (a)–(h) TG-DTG curves of the total iridoid extract, total flavonoid extract, total organic acid extract, geniposide (standard), chlorogenic acid, ursolic acid, GFPG powder, and GF; (i) TG curve of all samples; (j) DTG curve of all samples.

In the pyrolysis characteristic curve of each sample, the main pyrolysis stage of total iridoids for completely pyrolysis occurred at 225.7–351.6 °C (Table 1) and decreased in mass fraction by 34.71% (Fig 2a). The main pyrolysis stage of total flavonoids in GF was also observed at 162.1–346.9 °C with decrease of the mass fraction by 48.89% (Fig 2b), indicating that total flavonoids were pyrolysed in GF during pyrolysis stage. In combustion pyrolysis characteristic curve of total organic acids in GF, the main pyrolysis stage was observed at 87.8–411.2 °C and the mass fraction was reduced by 54.73% (Fig 2c). To compare the combustion and pyrolysis characteristic curves of geniposide, a main active compound of total iridoids in GF, we analysed a standard substance (Fig 2d). As the result, the main pyrolysis stage of geniposide was shown at 224.8–322.8 °C, and its mass fraction was decreased by 48.74%.

**Table 1:** Pyrolysis parameters of main compounds in GFPG.

Samples	Combustion heat stage	DTGmax%/min	Mass/%
Total Iridoids extract	Dehydration stage (ordinary–160.2 °C)	0.62	3.60
	(160.2 °C–225.7 °C)	3.99	14.22
	(225.0 °C–351.6 °C)	4.87	34.71
	(351.6 °C–565.3 °C)	1.21	13.25
Total flavonoids extract	Dehydration stage (ordinary–162.1 °C)	1.16	3.57
	(162.1 °C–346.9 °C)	5.22	48.89
	(346.9 °C–572.4 °C)	1.22	13.21
Total organic acids extract	Dehydration stage (ordinary–87.8 °C)	0.27	6.17
	(87.8 °C–411.2 °C)	3.36	54.73
	(411.2 °C–562.0 °C)	2.16	30.88
Geniposide (standard substance)	Dehydration stage (ordinary–224.8 °C)	0.18	2.45
	(224.8 °C–322.8 °C)	18.29	48.74
	(322.8 °C–537.1 °C)	9.45	45.25
Ursolic acid (standard substance)	Dehydration stage (ordinary–99.7 °C)	1.74	3.39
	(99.7 °C–170.7 °C)	1.85	4.33
	(170.7 °C–489.2 °C)	21.93	89.90
Chlorogenic acid (standard substance)	Dehydration stage (ordinary–59.7 °C)	0.65	0.89
	(59.7 °C–217.8 °C)	1.35	1.29
	(217.8 °C–402.6 °C)	5.21	45.52
	(402.6 °C–546.8 °C)	5.50	44.75
GF powder	Dehydration stage (ordinary–167.6 °C)	0.41	5.14
	(167.6 °C–216.2 °C)	3.26	6.24
	(216.2 °C–290.3 °C)	13.63	41.84
	(290.3 °C–403.4 °C)	10.28	16.16
GFPG test sample powder	Dehydration stage (ordinary–120.2 °C)	0.80	6.99
	(120.2 °C–208.0 °C)	2.01	6.04
	(208.0 °C–362.8 °C)	4.48	43.00
	(362.8 °C–527.3 °C)	7.97	37.54
	(412.4 °C–472.4 °C)	3.69	18.32

DTG, Max-maximum Thermogravimetric Rate; and Mass-thermogravimetric rate.

The main pyrolysis stage of chlorogenic acid in GF was shown at 217.8–402.6 °C with decrease in the mass fraction by 45.52%. In the characteristic combustion pyrolysis curves of its standard substance, its mass fraction was reduced by 1.29% at 59.7–217.8 °C in the partial pyrolysis stage (Fig 2e). In ursolic acid analysis, the mass fraction was reduced by 4.33% at 99.7–170.7 °C in the partial pyrolysis stage, and the main pyrolysis stage of its standard substance was shown at 170.7–489.2 °C, with decrease of its mass fraction by 89.89% (Fig 2f). By comparing total organic acids with pyrolysis parameters of chlorogenic acid and ursolic acid, it was inferred that this is the pyrolysis stage of various organic acids.

In the combustion and pyrolysis characteristics of GFPG test sample powder (Fig 2g) and GF powder (Fig 2h) with their main compounds, it can be inferred that organic acids such as total flavonoids, and other components were begun to pyrolyse in the range of 120.2–208.0 °C, with decrease of the mass fraction by 6.04%. It was decomposed first the total iridoids, organic acids, and total flavonoids in a range of 208.0–362.8 °C, with decrease of the mass fraction by 43.00%. This indicates that many active compounds in GF and GFPG begin to volatilise in this temperature range during pyrolysis stage.

Geniposide is the main active component of GFPG. Geniposide began to pyrolyse at 224.8°C. The TG (Fig 2i) and DTG (Fig 2j) data of all samples showed that some active components of ginger juice mixed with the GF powder also started to pyrolyse at 208–362.8 °C. Before reaching 208 °C, the main active components were dehydrated, the effective components remained relatively intact, and they started to lose mass at 208 °C. During GF processing with ginger, geniposide comprise the main effective component of GF and are retained to the greatest extent [19]. The bitterness and coldness of GF have been shown to improve the biological action of ginger. Corroborating these results, it was found that the best processing temperature of GFPG is 208 °C, the temperature which preserved the effective components well.

### Optimization of GFPG preparation process: single-factor testing and analysis

In our single-factor analysis, the contents of geniposide were 32.851, 34.308, 37.085, 27.432, and 16.745 mg/g at different solid-liquid ratios (w/v) with 10:1, 9:1, 8:1, 7:1, and 6:1, respectively. The highest content of geniposide was shown at a solid-liquid ratio of 8–1 (w/v). Also, at different processing time, 3, 4, 5, 6, 7, 8 min, its contents were 32.854, 31.833, 36.249, 18.472, 16.200, and 13.469 mg/g, respectively, and were remained the highest at 5 min. Meanwhile, the contents of geniposide were 25.597, 29.202, 29.493, 29.071and 28.377 mg/g at different moistening time of 1, 2, 3, 4, 5 h, respectively and were remained the highest at 3 h. From these results, it was determined optimal condition at 8:1 (w/v) for solid-liquid ratio, 5 min for processing time, and 3 h for moistening time.

### Optimization of GFPG preparation process: response surface test and analysis

**Fig 3:**
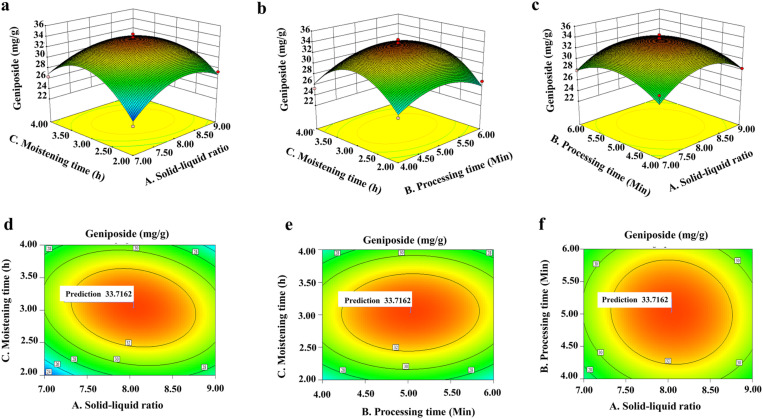
Response surface test for the interaction of various factors in GFPG processing. The 3D and planar plots show the interaction between solid-liquid ratio and moistening time **(a, d)**. The 3D and planar plots illustrate the interaction between processing time and moistening time **(b, e)**. The 3D and planar plots represent the interaction between solid-liquid ratio and processing time **(c, f)**.

It was presented the response surface models with the 3D and plan-view response surface diagrams of the interaction between the solid and liquid ratio, processing time, and moistening time on GFPG processing (Fig 3), and was shown data analysed by a Design-Export 8.0.6 program (Table 2). In this test, the parameters for optimal processing of GFPG were as follows: processing temperature, 208 °C; solid-liquid ratio (*A*), 8.01:1 w/v; processing time (*B*), 5.03 min; moistening time (*C*), 3.04 h; and the predicted geniposide content, 33.718 mg/g). In the corresponding quadratic equation model from the 3D model, we got the following results: *Y* = 33.70 + 0.33*A* + 0.16*B* + 0.40*C*-0.34*AB*-1.30 *AC* + 0.22*BC*-3.31*A*^*2*^-2.65*B*^*2*^-4.83*C*^*2*^ (*R*^*2*^ = 0.9454), indicating that this model has a good degree for fitting with a small test error and can use for analysis and prediction on processing.

**Table 2:** Results of response interview design with factor levels in the response surface test of GFPG.

Number	*A*	*B*/min	*C*/h	*Y (*mg·g^-1^)
1	8 (0)	5 (0)	3 (0)	33.94
2	9 (+1)	4 (−1)	3 (0)	28.30
3	8 (0)	5 (0)	3 (0)	34.47
4	8 (0)	5 (0)	3 (0)	33.84
5	7 (−1)	6 (+1)	3 (0)	27.88
6	7 (−1)	5 (0)	2 (−1)	22.64
7	7 (−1)	5 (0)	4 (+1)	26.37
8	8 (0)	5 (0)	3 (0)	32.31
9	9 (+1)	6 (+1)	3 (0)	26.40
10	7 (−1)	4 (−1)	3 (0)	28.40
11	8 (0)	5 (0)	3 (0)	33.94
12	9 (+1)	5 (0)	4 (+1)	25.89
13	9 (+1)	5 (0)	2 (−1)	27.34
14	8 (0)	6 (+1)	4 (+1)	27.58
15	8 (0)	4 (−1)	4 (+1)	25.31
16	8 (0)	4 (−1)	2 (−1)	25.30
17	8 (0)	6 (+1)	2 (−1)	26.69

Next, the influence of each factor on the variation of geniposide content in GFPG was investigated (Table 3), with the results indicating the following order of effect: processing time > solid-liquid ratio > moistening time. From the results for optimal conditions by using a software analysis, it was adjusted actual operation and optimal processing technology for GFPG preparation as follows: processing temperature, 208 °C; solid-liquid ratio, 8:1, w/v; processing time, 5 min; and moistening time, 3 h. At this condition, the content of geniposide in GFPG was 31.653 ± 0.577 mg/g. This was almost identical to the predicted value, indicating that RSM is good processing technique.

**Table 3:** Results of variance analysis in process optimization for GFPG.

Source	Sum of Squares	df	Mean Square	*F* Value	*P*-value Prob>F
Model	202.49	9	22.50	13.46	0.0012^**^
*A-Solid-liquid ratio*	0.87	1	0.87	0.52	0.4949
*B-Processing time*	0.20	1	0.20	0.12	0.7415
*C- Moistening time*	1.28	1	1.28	0.77	0.4105
*AB*	0.47	1	0.47	0.28	0.6132
*AC*	6.72	1	6.72	4.02	0.0851
*BC*	0.19	1	0.19	0.12	0.7438
*A* ^*2*^	46.10	1	46.10	27.58	0.0012^**^
*B* ^*2*^	29.59	1	29.59	17.70	0.0040^**^
*C* ^*2*^	98.37	1	98.37	58.84	0.0001^**^
Residual	11.70	7	1.67		
Lack of Fit	9.04	3	3.01	4.53	0.0891
Pure Error	2.66	4	0.66		
Cor Total	214.19	16			

### Analysis of active ingredients in ginger and GF on cholestatic hepatitis

**Fig 4:**
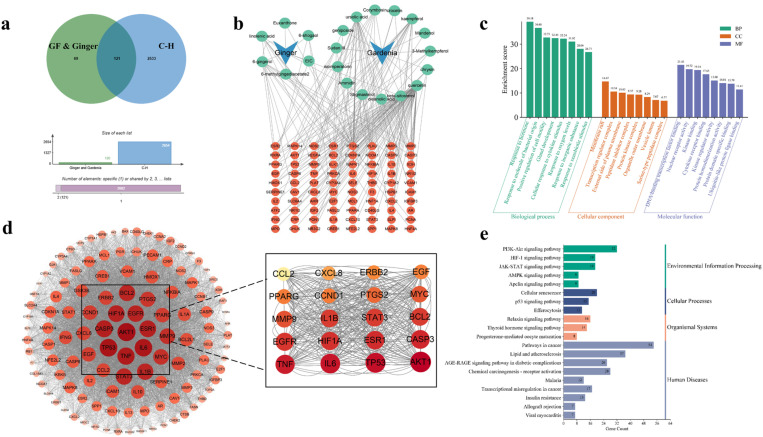
Network pharmacological analysis of GFPG on cholestatic liver injury. Map of the interaction targets of GFPG and C-H **(a)**. Interaction diagram between components and targets **(b)**. GO enrichment analysis **(c)**. Visualization of the target components with the interaction network **(d)**. KEGG pathway analysis **(e)**.

To identify the active ingredients-associated targets in ginger and GF, network pharmacological analysis was conducted. As the results, it was discovered 121 molecular targets for cholestatic hepatitis (Fig 4a).

### GO and KEGG analysis

Visualization of the network diagram for the active ingredients in ginger and GF, along with their common targets, was performed using Cytoscape software (Fig 4b). From the biological enrichment analysis of intersection targets by the Metascape database analysis, it was selected the top eight entries with visualisation plots (Fig 4c) as follows: “response to hormone,” “membrane raft,” and “DNA-binding transcription factor binding” for BP, CC, and MF, respectively.

Molecular targets related to cholestatic hepatitis from ginger and GF were imported into the STRING database to construct a PPI network and analyse target degree values. As the results, the circles representing targets were larger and darker that means more importance within the network (Fig 4d). The key targets of ginger and GF in cholestatic liver injury, identified by their highest degree values, included TNF-α, IL-6, AKT1, IL-1β, STAT3, CASP3, ESR1, HIF1A, and TP53.

KEGG pathway analysis showed that the active ingredients of ginger and GF in cholestatic hepatitis are closely involved in regulating pathways such as PI3K-AKT, HIF-1, JAK-STAT, AMPK, lipid metabolism, and atherosclerosis. (Fig 4e).

### Molecular docking analysis of GFPG active ingredients with key targets

**Fig 5:**
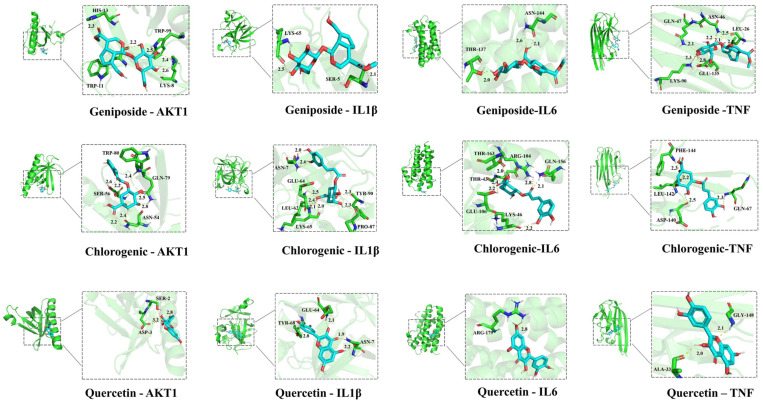
Molecular docking diagram of main components in GFPG with core molecular targets.

To explore the binding affinities of GFPG’s active components, including geniposide, chlorogenic acid, and quercetin, molecular docking was conducted with key PPI-identified targets AKT1, IL-6, IL-1β, and TNF-α (Fig 5). As the result, the binding energies of three compounds with four targets were less than −5 kJ/mol (Table 4). This is indicating that the therapeutic potential of GFPG on cholestatic hepatitis is from the good binding ability with core targets.

**Table 4:** Binding energy analysis of main components in GFPG with core molecular targets.

Target	PDB ID	Compound	Affinity (kcal/mol)
AKT1	1H10	Geniposide	−6.0
		Chlorogenic acid	−6.6
		Quercetin	−6.0
IL-1β	2NVH	Geniposide	−6.3
		Chlorogenic acid	−6.6
		Quercetin	−7.1
IL-6	1ALU	Geniposide	−5.9
		Chlorogenic acid	−6.4
		Quercetin	−7.0
TNF-α	5UUI	Geniposide	−6.6
		Chlorogenic acid	−6.4
		Quercetin	−7.0

### HPLC analysis of GF and GFPG extracts

In our HPLC analysis, the standard curves for main compounds, geniposide, chlorogenic acid, quercetin, and 6-gingerol were detected are shown in GF and GFPG extracts (S1 Table and S2 Table; S2 Fig.). The contents of chlorogenic acid, geniposide, and quercetin in GF extract were 5.72 ± 0.06, 113.53 ± 0.18, and 36.42 ± 1.52 μg/g, respectively. In GFPG extract, the contents of chlorogenic acid, geniposide, quercetin, and 6-gingerol were 5.24 ± 0.04, 97.73 ± 0.43, 58.10 ± 0.23, and 1.51 ± 0.01 mg/g, respectively.

### GFPG extract attenuates LPS- stimulated inflammation in RAW264.7 cells

**Fig 6:**
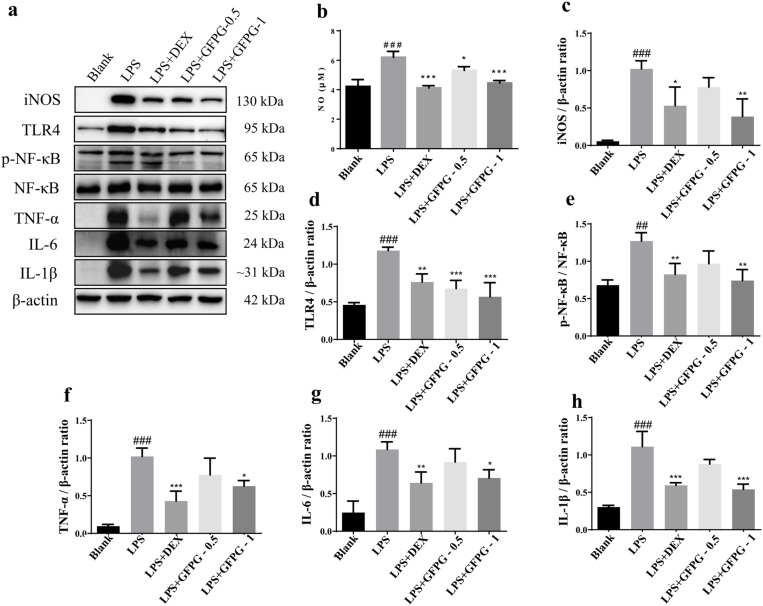
Effects of GFPG extract on LPS- stimulated inflammation in RAW264.7 cells. Protein expression levels of iNOS, TLR4, p-NF-κB, NF-κB, TNF-α, IL-6, and IL-1β were assessed by Western blotting **(a)**. NO concentrations in the culture medium were measured using the Griess assay **(b)**. The relative expression of iNOS **(c)**, TLR4 **(d)**, TNF-α **(f)**, IL-6 **(g)**, and IL-1β **(h)** was normalized to β-actin, while NF-κB phosphorylation was evaluated relative to total NF-κB **(e)**. Data are expressed as mean ± SD (*n* = 3). ^##^*P* < 0.05, ^##^*P* < 0.01, ^###^*P* < 0.001 vs. untreated control (Blank); ^*^P < 0.05, ^**^P < 0.01, ^***^P < 0.001 vs. LPS-treated group.

The anti-inflammatory effects of GFPG extract were assessed in LPS-stimulated RAW264.7 cells treated with varying concentrations. As the results, the production of inflammatory substance, NO was significantly (*P* < 0.001, Fig 6b) increased in LPS-stimulated cells compared to the normal cells with increase of the expression of inflammation mediators, iNOS (*P* < 0.001, Fig 6c), TLR4 (*P* < 0.001, Fig 6d), p-NF-κB (*P* < 0.01, Fig 6e), TNF-α (*P* < 0.001, Fig 6f), IL-6 (*P* < 0.001, Fig 6g), and IL-1β (*P* < 0.001, Fig 6h).

GFPG extract significantly reduced NO levels at 0.5 mg/mL (*P* < 0.05) and 1 mg/mL (*P* < 0.001) in LPS-stimulated cells. At 1 mg/mL, it also inhibited iNOS (*P* < 0.01), TLR4 (*P* < 0.001), TNF-α (*P* < 0.05), IL-1β (*P* < 0.001), IL-6 (*P* < 0.05), and NF-κB phosphorylation (*P* < 0.01). Similarly, dexamethasone (DEX) treatment markedly decreased NO levels (*P* < 0.001) and suppressed inflammatory mediators. These results suggest that GFPG extract effectively attenuates inflammation.

### Effects of GFPG extract on liver index and body weight

**Fig 7:**
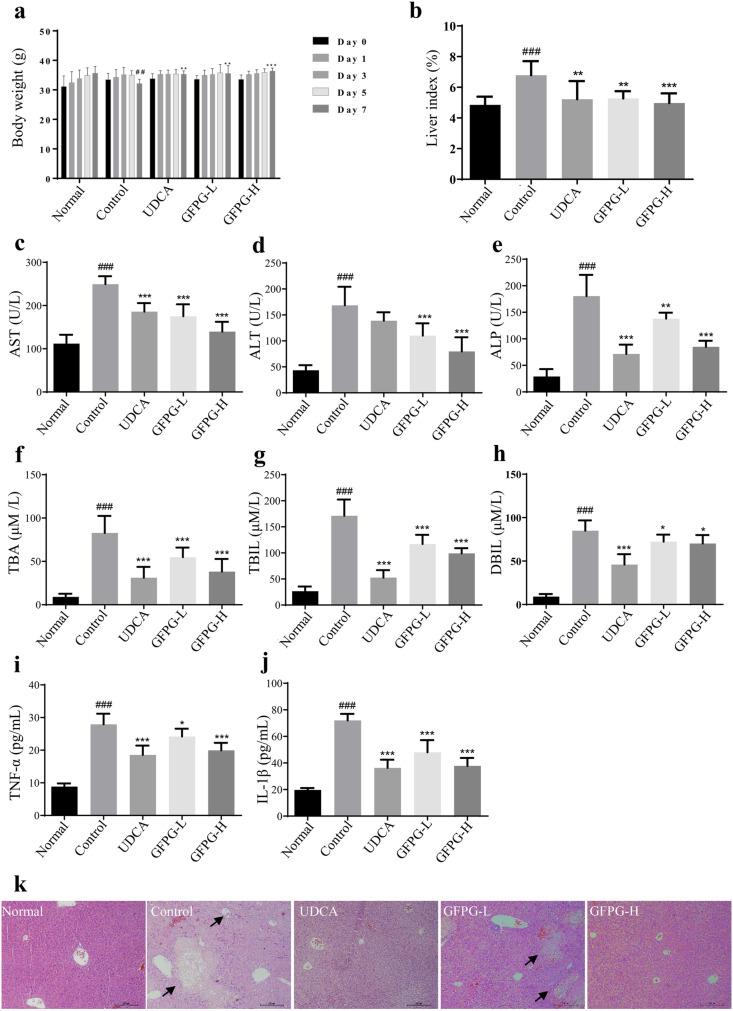
GFPG extract reduced the effects of ANIT-induced intrahepatic cholestasis in mice. The weights of body weight were measured once every two days **(a)**. Liver index of mice in each group **(b)**. Serum levels of each marker, including AST **(c)**, ALT **(d)**, ALP **(e)**, TBA **(f)**, TBIL **(g)**, DBIL **(h)**, TNF-α **(i)**, and IL-1β **(j)**, were measured in mice. The data are presented as mean ± SD (*n* = 8). ^#^*P* < 0.05, ^##^*P* < 0.01 and ^###^*P* < 0.001 compared to the normal group; ^*^P < 0.05, ^**^P < 0.01 and ^***^P < 0.001 compared to the control group. Representative hepatic H&E staining images (k) were observed under a microscope (×200), with areas of severe liver necrosis indicated by triangular arrows. GFPG-L, GFPG 250 mg/kg-administrated group; and GFPG-H, GFPG 500 mg/kg- administrated group.

ANIT-induce significantly reduced body weight in the control group (*P* < 0.01) compared to the normal group on day 7. However, GFPG-treated groups at both low (*P* < 0.01) and high (*P* < 0.001) doses showed significantly higher body weights than the control group (Fig 7a). The liver index was notably higher (*P* < 0.001) in the control group compared to the normal group, but GFPG administration significantly reduced the liver index at both doses (low: *P* < 0.01; high: *P* < 0.001) compared to the control group (Fig 7b). The UDCA-treated group also showed a significant reduction in liver index (*P* < 0.001). These results suggest that GFPG extract improves cholestatic liver injury.

### Effects of GFPG extract on the change of serological markers

Next, we measured the serological markers in the sera of the mice. The results demonstrated a significant increase in the levels of AST, ALT, ALP, TBA, TBIL, and DBIL in the control group (*P* < 0.001) compared to the normal group (Figs 7c-h). These results suggest that the cholestatic disease model was effectively created. The increasing levels of these markers were reduced significantly after administration of GFPG extract at low dose (*P* < 0.001 for AST, *P* < 0.001 for ALT, *P* < 0.01 for ALP, *P* < 0.001 for TBA, *P* < 0.001 for TBIL, and *P* < 0.05 for DBIL) and high dose (*P* < 0.001 for AST, *P* < 0.001 for ALT, *P* < 0.001 for ALP, *P* < 0.001 for TBA, *P* < 0.001 for TBIL, and *P* < 0.05 for DBIL) in cholestatic hepatitis mice. In UDCA-administrated group, it was also shown the significantly decrease of all markers (*P* < 0.001 for AST, *P* < 0.001 for ALP, *P* < 0.001 for TBA, *P* < 0.001 for TBIL, and *P* < 0.001 for DBIL) except ALT levels.

GFPG extract administration significantly reduced TNF-α and IL-1β levels at both low (*P* < 0.05 for TNF-α, *P* < 0.001 for IL-1β) and high doses (*P* < 0.001 for both) compared to the control group (Fig 7i, Fig 7j). UDCA treatment also led to significant decreases in TNF-α (*P* < 0.001) and IL-1β (*P* < 0.001) levels in cholestatic hepatitis mice. The findings suggest that GFPG extract protects against cholestatic liver damage by exerting anti-inflammatory effects and modulating bile acid metabolites in mice.

### Effects of GFPG extract on liver damage

To investigate the effect of GFPG extract on cholestatic liver damage, we observed the structural changes in liver tissues. In the normal group, it was observed an intact structure of liver cells with abundant cytoplasm, normal nuclei, and radially arranged hepatic cords extending from the central vein (Fig 7k). In contrast, the control group exhibited focal necrosis of liver cells, loose connective tissue, and extensive infiltration of inflammatory cells. GFPG administration at low and high doses alleviated histological changes, including reduced inflammatory infiltration, localized necrosis, preserved cellular structure, and well-organized hepatic cords. It was also observed in UDCA-administrated group that liver cells were tightly arranged with minimal inflammatory infiltration.

### Effects of GFPG extract on TLR4/NF-κB and FXR/PPAR-α signaling pathways in liver tissues

**Fig 8:**
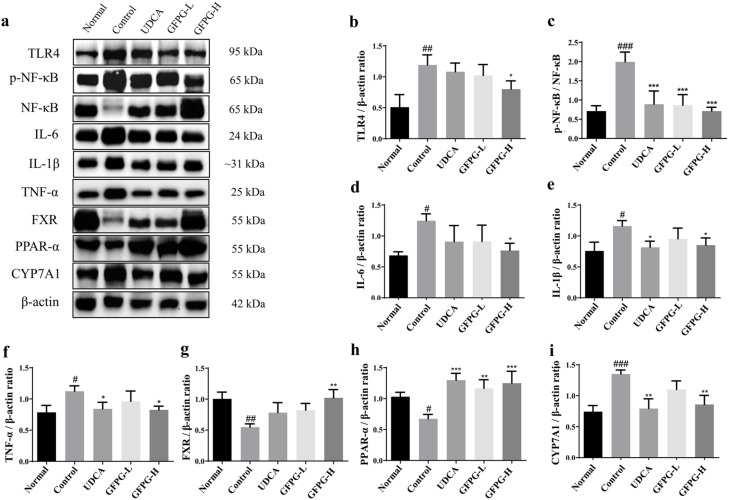
The effect of GFPG extract on the TLR4/NF-κB and FXR/PPAR-α signaling pathways in liver tissue of mice with cholestatic liver injury. Expressions of TLR4, p-NF-κB, NF-κB, IL-6, IL-1β, TNF-α, FXR, PPAR-α and CYP7A1 were determined by western blotting **(a)**. The histograms from **b** to **i** were calculated relatively for the expression of each target compared with β-actin or NF-κB (for p-NF-κB). Data are expressed as means ± standard deviations. ^#^*P* < 0.05 ^##^*P* < 0.01 and ^###^*P* < 0.001 vs. normal group, ^*^P < 0.05 ^**^P < 0.01 and ^***^P < 0.001 vs. control group.

To identify the pharmacological mechanism of GFPG extract on cholestatic liver damage, it was investigated the signalling pathways related with cholestatic hepatitis in liver tissues. To elucidate the pharmacological mechanisms underlying the effects of GFPG extract on cholestatic liver injury, we examined the signaling pathways involved in cholestatic hepatitis in liver tissues. In the control group, the expression of TLR4, NF-κB, IL-6, IL-1β, TNF-α, and CYP7A1 proteins was significantly elevated compared to normal levels (*P* < 0.05, *P* < 0.01, *P* < 0.001), whereas the expression of FXR and PPAR-α was notably downregulated (*P* < 0.05, *P* < 0.01). Treatment with GFPG extract (GFPG-H group) resulted in a significant reduction in the expression levels of TLR4, p-NF-κB/NF-κB, IL-6, IL-1β, TNF-α, and CYP7A1 proteins (*P* < 0.05, *P* < 0.01, *P* < 0.001), accompanied by a substantial increase in the expression of FXR and PPAR-α (*P* < 0.01, *P* < 0.001). The detailed results are illustrated in Fig 8.

### Effects of GFPG extract on the PI3K/AKT/GSK-3β signaling pathway in mice with cholestatic liver injury

**Fig 9:**
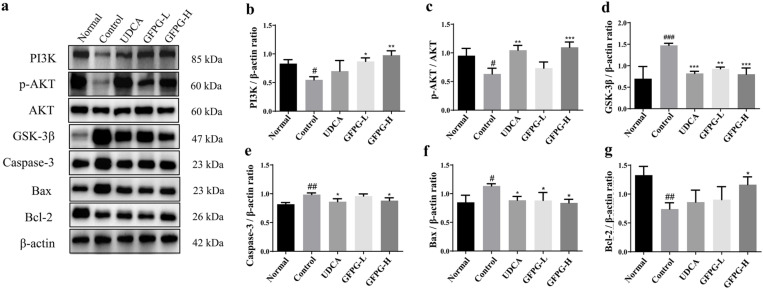
GFPG extract’s effect on the PI3K/AKT/GSK-3β signaling pathway in the liver tissue of mice with cholestatic liver injury.

Compared to the normal group, the cholestasis model group showed significantly reduced PI3K, p-AKT/AKT, and Bcl-2 expression (*P* < 0.05, *P* < 0.01), while GSK-3β, caspase-3, and Bax levels were significantly increased (*P* < 0.05, *P* < 0.01, *P* < 0.001). In the GFPG-H group, PI3K, p-AKT/AKT, and Bcl-2 levels were significantly higher (*P* < 0.05, *P* < 0.01, *P* < 0.001), while GSK-3β, caspase-3, and Bax expression was significantly lower (*P* < 0.05, *P* < 0.001) compared to the ANIT-induced cholestasis group. Results are shown in Fig 9.

The expression levels of PI3K, AKT, p-AKT, GSK-3β, caspase-3, Bcl-2, and Bax were measured by Western blotting (a). The histograms (b–g) represent the relative expression of each target, normalized to β-actin or AKT (for p-AKT). Data are presented as means ± standard deviations. ^#^*P* < 0.05, ^##^*P* < 0.01, and ^###^*P* < 0.001 compared to the normal group; ^*^P < 0.05, ^**^P < 0.01, and ^***^P < 0.001 compared to the control group.

## Discussion

Official standards for processing of Chinese herbal medicines such as in Beijing, Tianjin, and Fujian include records of the processing technology of GFPG. Nevertheless, there are still different technology from each other, e.g., no specific temperature and processing time for standardization that is affecting the clinical application of GFPG. Therefore, it is necessary to optimise the processing condition of GFPG preparation for clinical applications or experimental studies. Recently, it has been gradually applied herbal processing techniques such as a thermal analysis [20]. In this study, it was performed a thermogravimetry (TG) and differential thermal analysis (DTG) to determine the best processing temperature of GFPG and was applied RSM technique to optimise the optimal conditions for the herbal mixes, such as the ratio of ginger juice to GF and the time for moistening and processing. In our analysis, to optimize the processing condition of GFPG, it was also objectively optimised by combining thermal analysis and RSM. It was difficult to comprehensively identify the specific compounds of GFPG owing to the intricate composition of herbal substances. Therefore, our procedure for thermal analysis was focused on determining the pyrolysis characteristic curves of the main components of GF, such as total iridoids, flavonoids, and organic acids. A thermogravimetry is an objective and accurate method for studying the pyrolysis characteristics of various active components of herbal extracts. In this study, we also used a TG technology during the thermal processing of GF with ginger juice and identified the characteristics of the internal material pyrolysis of GFPG.

In the concept of TCM, it is well-known that the four herbal characteristics–cold, heat, warm, and cool–can assist in the regulation of abnormal body conditions. Gardenia Fructus is a cold herb, therefore, it is believed that the use of its raw materials induces digestive problems such as strong gastric irritation. However, from the “taking medicine and pharmacy concept” in TCM, GF is commonly used in clinics after changing its “cold” character to “warm” upon processing with ginger, a representative herb with a strong warm property [21]. In addition, the change in the herbal nature via processing can help increase the efficiency of herbal therapy. From this principle, we applied herbal processing techniques such as a thermal analysis and RSM for GFPG preparation.

Geniposide, the primary active compound in both GF and GFPG, possesses a range of medicinal properties, including hepatoprotective, neuroprotective, anti-diabetic, and anti-inflammatory effects [22]. During the processing of GF with ginger juice, heating the GF peel influenced the dissolution of geniposide. In this study, we analysed the content of geniposide as an indicator of herbal changes after processing. In the HPLC analysis, three compounds, geniposide, chlorogenic acid, and quercetin were identified as the main compounds in GF and GFPG. The detection of geniposide and chlorogenic acid ensures quality control and consistency among replicated samples. Quercetin, identified as a core component through network pharmacology analysis, though not species-specific, provides complementary insights into the multi-target mechanisms of GFPG. After processing, a slight reduction in chlorogenic acid was observed; however, no significant difference was noted in the contents of geniposide and quercetin in GFPG. This finding suggests that the processing of GF with ginger juice does not affect the main compounds of GF, especially geniposide.

Recently, network pharmacology analysis has been used to elucidate the molecular mechanisms underlying the pharmacological actions of herbal medicines. Analysis of various molecular networks with complex multi-layered interactions can intuitively demonstrate the relationship between ingredients and the pharmaceutical mechanisms of herbs and rapidly discover potential therapeutic targets of herbs and their effective components [23]. Additionally, molecular docking analysis models the interactions between herbs and molecular targets, determining the binding affinity of the compounds. These analytical methods are valuable for validating network pharmacology results. In this study, we analysed the molecular targets of GFPG related to its therapeutic effects in cholestatic hepatitis using network pharmacology and molecular docking. The key targets identified were TNF-α, IL-6, AKT1, and IL-1β, which are involved in the PI3K/AKT and AMPK pathways. Molecular docking showed that these targets bind favorably with the main compounds of GFPG. These findings suggest that GFPG’s effects on cholestatic hepatitis are associated with inflammation regulation.

In RAW264.7 cells, GFPG extract inhibited the TLR4/NF-κB pathway, reducing LPS-induced NO production and the expression of iNOS, TNF-α, IL-6, and IL-1β. It is well-known that NO is a critical regulatory factor in intercellular communication, but its excessive production stems from the expression of iNOS and NO synthase activity that induces inflammatory response in cells [24]. Toll-like receptors (TLRs) are essential pattern recognition receptors that are pivotal in initiating inflammation and shaping the immune response. Activation of the NF-κB pathway triggers the release of pro-inflammatory cytokines like TNF-α, IL-6, and IL-1β, contributing to the pathogenesis of hepatic inflammatory diseases, including cholestatic hepatitis [25]. In our study, GFPG administration significantly reduced LPS-induced inflammatory protein expression in RAW 264.7 cells, suggesting that GFPG suppresses inflammation by modulating the TLR4/NF-κB pathway.

In our *in vivo* study, a cholestatic hepatitis mouse model was established using ANIT induction. ANIT is a well-recognized hepatotoxic chemical that can mimic cholangitis, characterized by cholestasis accompanied by a bile duct obstruction with hepatocyte necrosis [26], so, it has been used for studying cholestatic hepatitis. The extent of hepatocyte injury can be reflected by increase of ALT and AST levels in the serum. When cell membranes are damaged, leading to increased permeability, ALT and AST leak from the cells and mitochondria into the bloodstream. Therefore, higher serum levels of ALT and AST indicate greater degrees of cellular damage or necrosis. Meanwhile, ALP levels reflect impaired bile flow, while total bilirubin (TBIL) and direct bilirubin (DBIL) levels indicate directly hepatic metabolic status [27]. In this study, GFPG administration significantly reduced serum ALT, AST, ALP, TBA, TBIL, and DBIL levels in mice with ANIT-induced cholestatic hepatitis. It also alleviated weight loss and decreased the liver index following ANIT treatment.

Cytotoxicity and the release of inflammatory mediators are key factors in ANIT-induced liver injury [28]. NF-κB regulates the translocation of inflammatory signals, triggering the expression of IL-1β, IL-6, and TNF-α in hepatocytes [29]. These inflammatory factors can lead to a sustained decrease in bile acid transporter levels, thereby promoting the accumulation of bile acids and exacerbating cholestasis [30]. In our study, the expression of inflammatory factors, including TLR4, NF-κB, IL-6, IL-1β, and TNF-α, was elevated in liver tissues, along with increased serum levels of TNF-α and IL-1β in mice with ANIT-induced cholestatic hepatitis. The findings suggest that GFPG alleviates ANIT-induced cholestatic liver damage by regulating excessive inflammation.

The farnesoid X receptor (FXR) is the primary bile acid sensor and functions as a transcription factor activated by bile acids [31]. Recent research has established PPARα as a key regulator of bile acid metabolism [32]. Agonists of PPARα such as fibrates, act as adjunctive therapies that can mitigate cholestasis-related diseases by modulating bile acid synthesis, transport, and other processes [33]. Additionally, PPARα is a nuclear receptor protein and regulates the stability of lipids and glucose while alleviating inflammatory responses [34]. PPARα inhibits pro-inflammatory cytokine production, including TNF-α and IL-1β, reducing liver inflammation [35]. The FXR pathway regulates bile acid synthesis, with CYP7A1 as the rate-limiting enzyme. In ANIT-induced cholestatic hepatitis mice, GFPG administration alleviates the suppression of FXR and PPARα expression, suggesting that GFPG inhibits bile acid synthesis, promotes bile flow, and reduces CYP7A1 expression. Additionally, GFPG suppresses TLR4/NF-κB activation, highlighting its potential to protect against cholestatic liver injury and alleviate bile accumulation.

In human cells, the PI3K/AKT/GSK-3β signaling pathway is a key regulator of processes such as cell growth, differentiation, metabolism, and apoptosis [36]. In particular, the PI3K as an essential intracellular signaling molecule [37], activated and subsequently activates downstream factor, AKT. GSK-3β is known to enhance the expression of pro-apoptotic factors, including caspase-3 and Bax, while suppressing the expression of the anti-apoptotic factor Bcl-2 [38]. The AKT inhibits expression of the downstream molecule, GSK-3β, thereby suppressing the apoptotic process in hepatocytes. In this study, the expression of PI3K, AKT, and Bcl-2 was suppressed in liver tissues of ANIT-induced cholestatic hepatitis mice, but the expression of GSK-3β, caspase-3, and Bax increased. This result indicates that ANIT injection induces apoptosis of liver cells. However, administration of GFPG extract significantly improved the expression levels of these targets compared to the ANIT-treated group, suggesting that GFPG extract inhibits apoptosis of hepatocytes in mice with cholestatic hepatitis.

## Conclusion

In our study, the optimal processing condition for GFPG preparation was determined from the thermal analysis combined with the response surface method. It was simulated and analysed the heating process during the GFPG processing and optimised the processing technology. *In vitro* and *in vivo* efficacy studies show that GFPG extract mitigates ANIT-induced cholestatic liver injury in mice by regulating bile acid metabolism and inhibiting inflammation and apoptosis. The mechanisms underlying the actions of GFPG extract likely involve the regulation of the TLR4/NF-κB and FXR/PPAR-α/GSK-3β signalling pathways within the livers of cholestatic hepatitis mice.

## Supporting information

S1 Fig Effect of GFPG on survival rate of RAW 264.7 cells. (TIF)

S2 Fig HPLC chromatogram for geniposide, chlorogenic acid, quercetin, and 6-gingerol. (TIF)

S3 Fig Graphical abstract. (TIF)

S1 Table The standard curve results for geniposide, chlorogenic acid, quercetin, and 6-gingerol. (DOCX)

S2 Table Sample results for geniposide, chlorogenic acid, quercetin, and 6-gingerol. (DOCX)

S1 File Supplement. (DOCX)

S2 File The original strip map of western blot. (PDF)

S3 File Supporting data for the chart. (XLSX)

## Abbreviations

ALP: Alkaline phosphatase
ALT: alanine aminotransferase
ANIT: alpha-naphthylisothiocyanate
AST: aspartate aminotransferase
BP: biological processes
CC: cellular components
CYP7A1: cholesterol 7α-hydroxylase
C-H: cholestatic hepatitis
DEX: dexamethasone
DTG: derivative thermogravimetry
DTGmax: maximum thermogravimetric rate
DBIL: direct bilirubin
DL: drug-likeness
FBS: foetal bovine serum
FXR: farnesoid X receptor
GF: Gardeniae Fructus
GFPG: Gardeniae Fructus processed with ginger juice
HE: haematoxylin and eosin
HPLC: high-performance liquid chromatography
iNOS: inducible nitric oxide synthase
IL-1β: interleukin-1 beta
IL-6: interleukin-6
MF: molecular functions
NF-κB: nuclear factor-κB
OB: oral bioavailability
PPAR-α: peroxisome proliferator activated receptor α
PPI: protein-protein interaction
PI3K: phosphatidylinositol-3-kinase
RSM: response surface methodology
TBA: total bile acids
T-BIL: total bilirubin
TG: thermogravimetry
TCMSP: traditional chinese medicine systems pharmacology database and analysis platform
TNF-α: tumour necrosis factor-alpha
TCM: Traditional Chinese Medicine
TLR4: toll-like receptor 4
UDCA: ursodeoxycholic acid.
