# Supplementary figures and images for: Optimized processing of Gardenia Fruits with ginger juice: Unveiling therapeutic mechanisms for cholestatic liver injury through TLR4/NF-κB, FXR/PPAR-α, and PI3K/AKT/GSK-3β
Source: PLoS One. 2025 Sep 16;20(9):e0330189. doi: 10.1371/journal.pone.0330189 (PMC12440179; doi:10.1371/journal.pone.0330189)

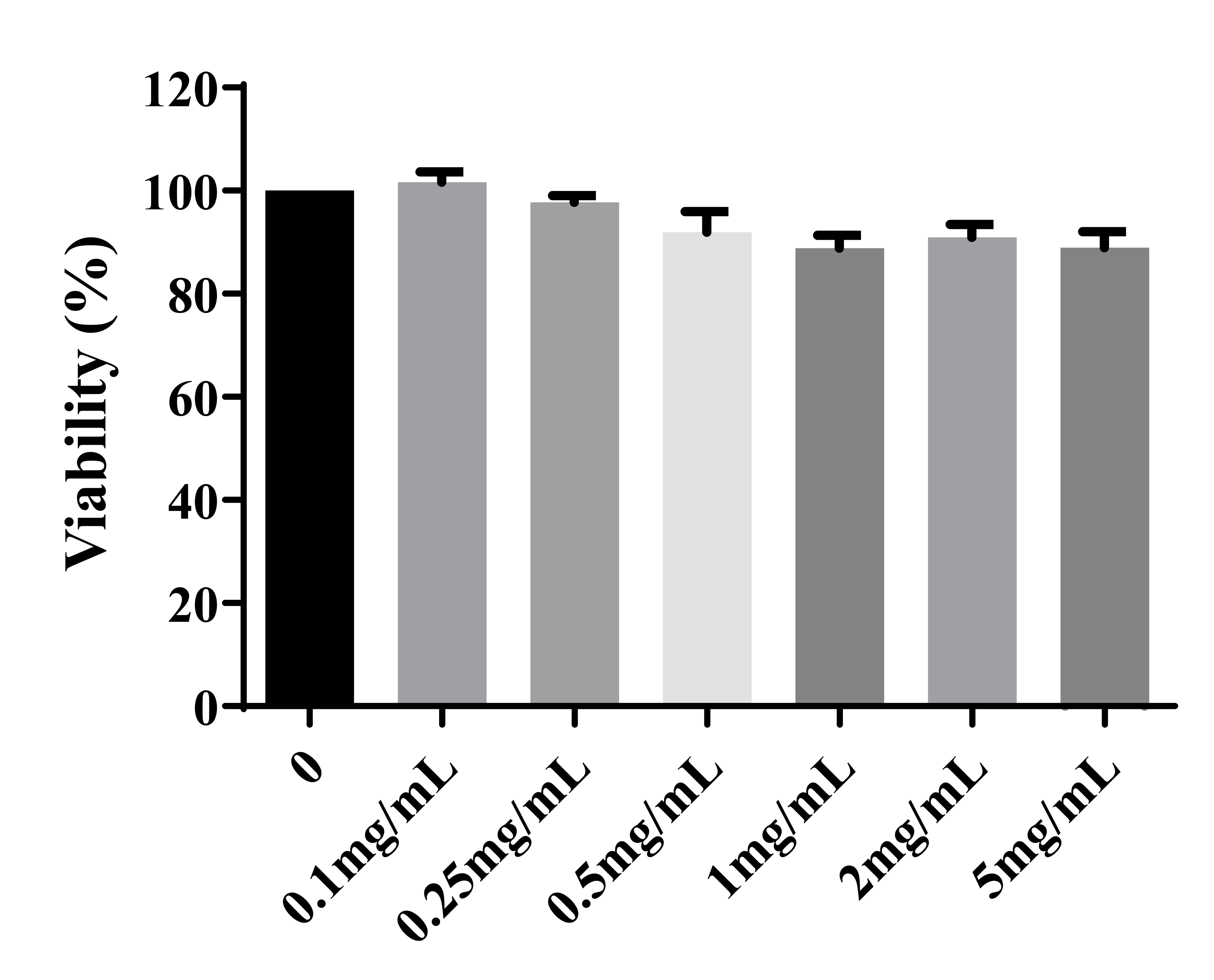

Supplement: S1 Fig — (TIF) [file pone.0330189.s001.tif]

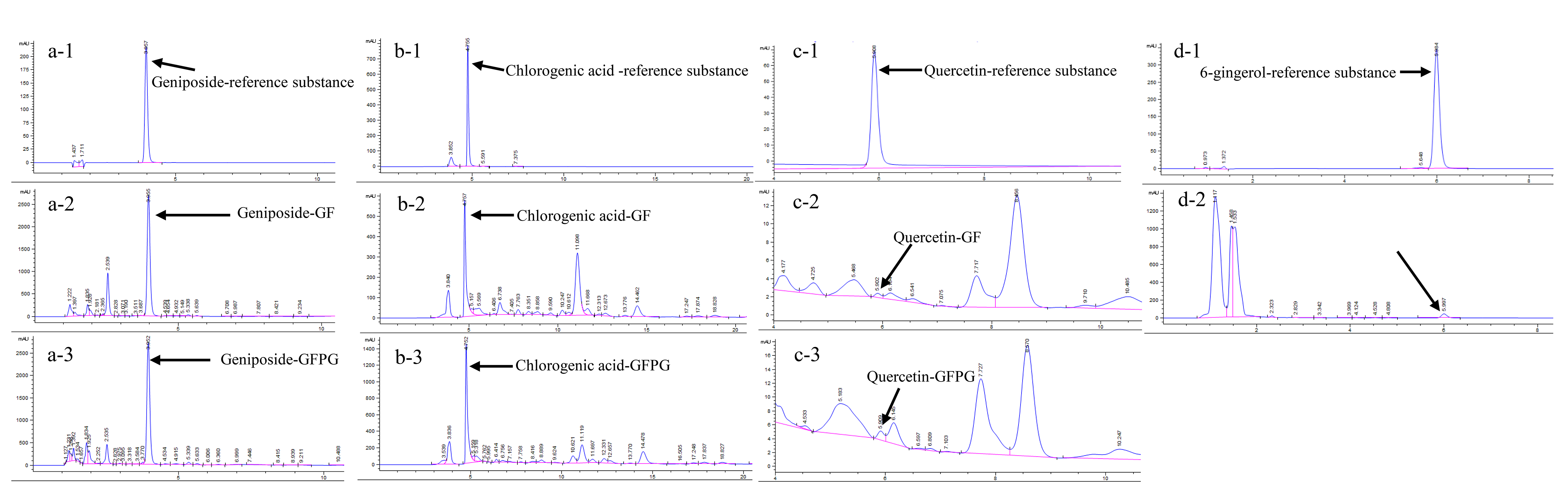

Supplement: S2 Fig — (TIF) [file pone.0330189.s002.tif]

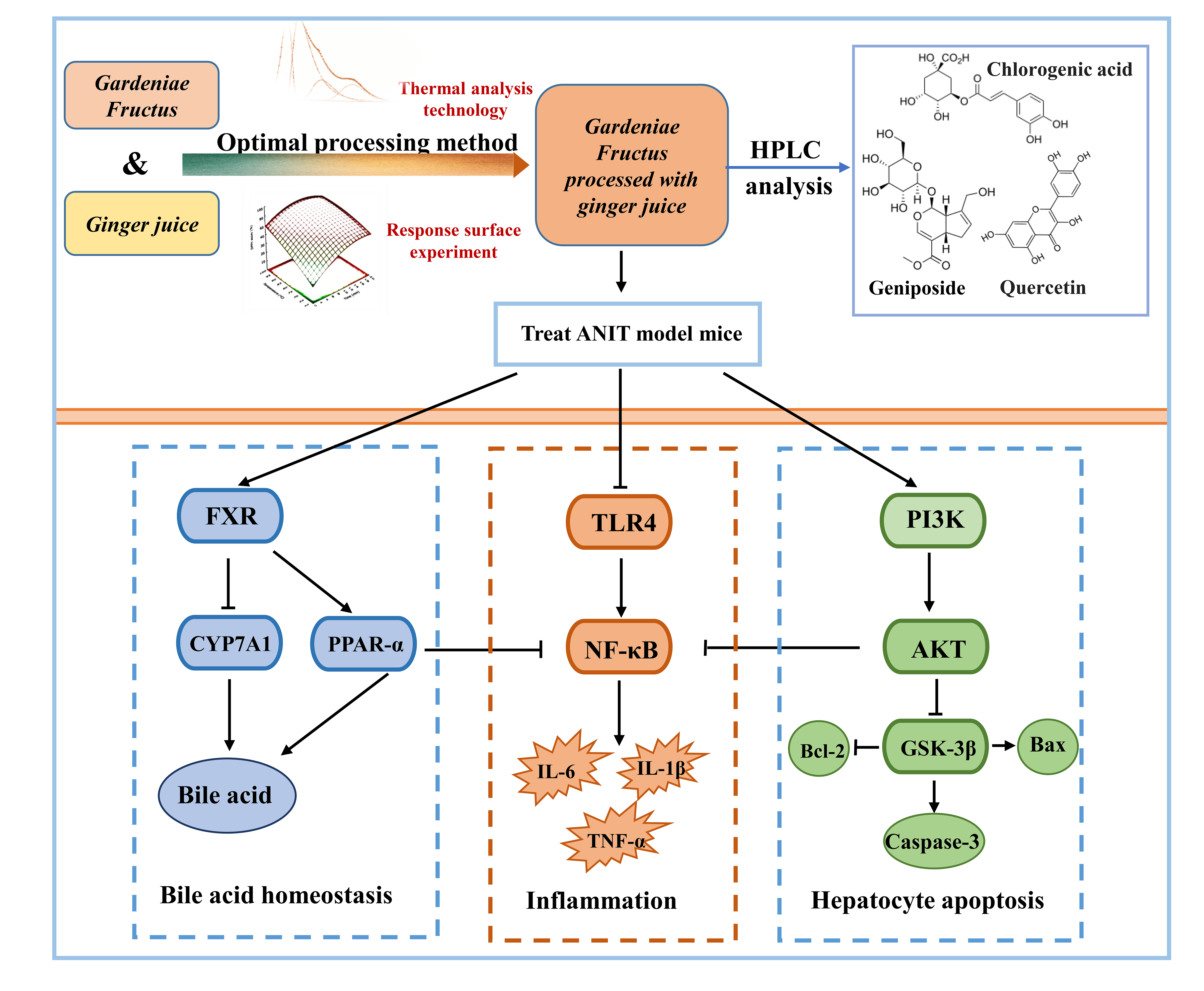

Supplement: S3 Fig — (TIF) [file pone.0330189.s003.tif]
