# Supplementary material for: Optimized processing of Gardenia Fruits with ginger juice: Unveiling therapeutic mechanisms for cholestatic liver injury through TLR4/NF-κB, FXR/PPAR-α, and PI3K/AKT/GSK-3β
Source: PLoS One. 2025 Sep 16;20(9):e0330189. doi: 10.1371/journal.pone.0330189 (PMC12440179; doi:10.1371/journal.pone.0330189)
Supplement: S1 Table — (DOCX) [file pone.0330189.s004.docx]

**S1 Table**. The standard curve results for geniposide, chlorogenic acid, quercetin, and 6-gingerol

| Reference Substance | Standard curve equation | *r*^2^ | Range of linearity |
| --- | --- | --- | --- |
| Geniposide | *y*=7.388*x*-3.8761 | 0.9999 | 205.0μg/mL~12.8μg/mL |
| Chlorogenic acid | *y*=38.646*x*+365.02 | 0.9999 | 255.0μg/mL ~ 15.94μg/mL |
| Quercetin | *y*=18.326*x*-11.899 | 0.9999 | 290.0μg/mL ~ 18.125μg/mL |
| 6-gingeror | *y=11.673x+51.003* | 1 | 1000μg/mL ~ 62.5μg/mL |
