# Supplementary material for: Optimized processing of Gardenia Fruits with ginger juice: Unveiling therapeutic mechanisms for cholestatic liver injury through TLR4/NF-κB, FXR/PPAR-α, and PI3K/AKT/GSK-3β
Source: PLoS One. 2025 Sep 16;20(9):e0330189. doi: 10.1371/journal.pone.0330189 (PMC12440179; doi:10.1371/journal.pone.0330189)
Supplement: S2 Table — (DOCX) [file pone.0330189.s005.docx]

**S2 Table**. Sample results for geniposide, chlorogenic acid, quercetin, and 6-gingerol

| Reference Substance | Sample | 1 | 2 | 3 | Mean | SD | Unit |
| --- | --- | --- | --- | --- | --- | --- | --- |
| Chlorogenic acid | GF | 5.66 | 5.72 | 5.77 | 5.72 | 0.06 | mg/g |
|  | GFPG | 5.24 | 5.28 | 5.20 | 5.24 | 0.04 |  |
| Geniposide | GF | 113.37 | 113.72 | 113.51 | 113.53 | 0.18 | mg/g |
|  | GFPG | 98.09 | 97.85 | 97.26 | 97.73 | 0.43 |  |
| Quercetin | GF | 34.80 | 36.65 | 37.81 | 36.42 | 1.52 | ug/g |
|  | GFPG | 57.97 | 58.36 | 57.97 | 58.10 | 0.23 |  |
| 6-gingerol | GFPG | 1.50 | 1.51 | 1.51 | 1.51 | 0.01 | mg/g |
