# Supplementary material for: Optimized processing of Gardenia Fruits with ginger juice: Unveiling therapeutic mechanisms for cholestatic liver injury through TLR4/NF-κB, FXR/PPAR-α, and PI3K/AKT/GSK-3β
Source: PLoS One. 2025 Sep 16;20(9):e0330189. doi: 10.1371/journal.pone.0330189 (PMC12440179; doi:10.1371/journal.pone.0330189)
Supplement: S1 File — (DOCX) [file pone.0330189.s006.docx]

**1. Optimization of the Processing Technology of Gardeniae Fructus processed with ginger juice (GFPG) by Response Surface Methodology (RSM)**

*Determination of* *geniposide*

To make standard solution, geniposide was solved in 100% methanol at 30 μg/1 mL. The test sample was prepared by taking about 0.1 g of GFPG sample powder, weighing it accurately, putting it into a conical flask with a stopper, followed by addition of 25 mL methanol precisely, weighing it, treating it ultrasonically for 20 min, cooling it down, weighing it again, making up the lost weight with methanol, and shaking well and then filtering. 10 mL of the filtrate was accurately measured, placed in a 25 mL volumetric flask, methanol was added to the scale, shake well, and then prepared.

High-performance liquid chromatography (HPLC, Thermo Fisher Scientific U3000) was used with Tnature-C_18_ (250 mm×4.6 mm, 5 μm) column, with a volume flow rate of 1 mL/min, column temperature of 25℃, mobile phase of acetonitrile water (15:85, v/v), and detected at 238 nm. Appropriate amount of geniposide was accurately weighed, placed it in a 25 mL volumetric flask, and the geniposide solution at 0.5 mg·mL^-1^ was prepared as a standard solution.

Take 1, 2, 3, 4, and 6 mL of geniposide solution into a 10 mL volumetric flask and fix the volume at 0.05 mg·mL^-1^, 0.1 mg·mL^-1^, 0.15 mg·mL^-1^, 0.2 mg·mL^-1^, and 0.3 mg·mL^-1^, respectively. The linear range was calculated the peak area integral value as the ordinate (*Y*) and the mass concentration as the abscissa (*X*).

*Results of geniposide determination*

The regression equation, correlation coefficient (*r*) and the linear range were *Y* = 160.88*X*+1.9639 *r^2^*=0.9999, and the linear range was 0.05~0.3 mg/mL. The RSD of precision test, stability test, repeatability test and recovery test were 0.84%, 0.94%, 1.15% and 2.41%, respectively. The method was shown to be good and feasible.

**2. Determination of cell toxicity of Gardeniae Fructus processed with ginger juice (GFPG) extract on RAW 264.7 cell**

Macrophages in optimal growth conditions were selected and seeded into a 96-well plate at a density of 1.5×10^4^ cells per well. After allowing 24 hours for cell growth and adhesion, various concentrations of GFPG extract (0, 0.1, 0.25, 0.5, 1, 2, and 3 mg/L) were added. The cells were then incubated for an additional 12 hours. The cell viability measured by cell viability assay kit (cat no. K47042702, DoGenBio Co., Korea). The 3-(4,5-dimethylthiazol-2-yl)-2,5-diphenyl tetrazolium bromide solution (20 g/mL) was added to each well and incubated for 2 h, and the absorbance was measured at 450 nm using a microplate reader (Molecular Devices, USA). Cell viability was calculated as a percentage of untreated cells. Hence, GFPG extract at 0.1–2 mg/mL did not demonstrate significantly decreased cell viability. Therefore, we used three concentrations of the *GFPG* extract (0.5 and 1 mg/mL) for the efficacy test (S1 Fig).

The formula for calculating cell viability is as follows:

Viability (%) = [(OD_2_-OD_0_)/(OD_1_-OD_0_)] × 100%

where OD_0_ represents the optical density of the blank well, OD_1_ represents the optical density of the control well, and OD_2_ represents the optical density of the experimental well.

**S1 Fig.** Effect of GFPG on survival rate of RAW 264.7 cells (*n*=3)

**3.** **Determination of main compounds in Gardeniae Fructus (GF),** **Gardeniae Fructus processed with ginger juice (GFPG) extract by high-performance liquid chromatography (HPLC) analysis**

*3.1. Chromatographic conditions*

**Chlorogenic acid**: For the analysis, an Agilent ZORBAX Eclipse XDB-C18 column (Analytical 4.6 × 150 mm 5-micron) on Agilent Technologies 1260 Infinity II HPLC system and isometric elution using methyl alcohol (23%) and 0.1% phosphoric acid water (77%) as mobile phases were used. The flow rate was 1.0 mL/min with a column temperature of 40 ℃. The detection wavelength of the UV detector was set at 321 nm.

**Geniposide**: For the analysis, an Agilent ZORBAX Eclipse XDB-C18 column (Analytical 4.6 × 150 mm 5-Micron) on Agilent Technologies 1260 Infinity II HPLC system and isometric elution using acetonitrile (15%) and water (85%) as mobile phases were used. The flow rate was 1.0 mL/min with a column temperature of 30 ℃. The detection wavelength of the UV detector was set at 238 nm.

**Quercetin**: For the analysis, an Agilent ZORBAX Eclipse XDB-C18 column (Analytical 4.6 × 150 mm 5-Micron) on Agilent Technologies 1260 Infinity II HPLC system and isometric elution using acetonitrile (30%) and water (70%) as mobile phases were used. The flow rate was 1.0 mL/min with a column temperature of 30 ℃. The detection wavelength of the UV detector was set at 370 nm.

**6-gingeror:** For the analysis, an Agilent ZORBAX Eclipse XDB-C18 column (Analytical 4.6 × 150 mm 5-Micron) on Agilent Technologies 1260 Infinity II HPLC system and isometric elution using acetonitrile (48%) and water (52%) as mobile phases were used. The flow rate was 1.0 mL/min with a column temperature of 25 ℃. The detection wavelength of the UV detector was set at 226 nm.

*3.2. Preparation of standard and sample solutions*

An accurate amount of chlorogenic acid was precisely weighed and transferred into a 10 mL volumetric flask. Subsequently, 70% methanol was added to dissolve the compound, and the solution was then diluted to the 10mL. After thorough mixing, a reference substance stock solution containing 255.0 μg/mL of chlorogenic acid was prepared. Similarly, geniposide was meticulously weighed and placed into a 10 mL volumetric flask. Following the addition of 70% methanol and subsequent dilution to the 10 mL, the solution was vigorously shaken to produce a reference substance stock solution with a concentration of 205 μg/mL of geniposide. Quercetin was accurately weighed and transferred into a 10 mL volumetric flask, followed by the addition of 70% methanol. The solution was then diluted to the 10mL and thoroughly mixed, resulting in a reference substance stock solution containing 290 μg/mL of quercetin. Likewise, 6-gingerol reference standard was precisely weighed and transferred into a 10 mL volumetric flask. After adding 70% methanol and diluting to the 10mL, the solution was shaken, obtained a reference substance stock solution with a concentration of 1000 μg/mL of 6-gingerol.

Approximately 0.25 g each of Gardeniae Fructus (GF) extract, and Gardeniae Fructus processed with ginger (GFPG) extract were accurately weighed and placed into separate conical flasks. Subsequently, 50 mL of 70% methanol was precisely added to each flask, which was then tightly sealed. The flasks underwent ultrasonication at 500W and 40kHz for 5 minutes, followed by cooling. The resultant solutions underwent centrifugation at 13000 rotations per minute (rpm) for 10 minutes to facilitate the separation of supernatant, which was subsequently passed through a 0.22 μm syringe filter to ensure particle-free quality for subsequent applications.

*3.3. Standard curve preparation*

An appropriate volume of the geniposide stock solution at a concentration of 205 μg/mL was serially diluted to prepare reference substance solutions with the following concentrations: 205 μg/mL, 102.5 μg/mL, 51.25 μg/mL, 25.63 μg/mL, and 12.8 μg/mL. Similarly, the chlorogenic acid stock solution at a concentration of 255.0 μg/mL was serially diluted to yield reference substance solutions with concentrations of 255.0 μg/mL, 127.5 μg/mL, 63.75 μg/mL, 31.88 μg/mL, and 15.94 μg/mL. The quercetin stock solution at a concentration of 290.0 μg/mL was also serially diluted to produce reference substance solutions with concentrations of 290.0 μg/mL, 145 μg/mL, 72.5 μg/mL, 36.25 μg/mL, and 18.125 μg/mL. Finally, the 6-gingerol stock solution at a concentration of 1000 μg/mL was serially diluted to obtain reference substance solutions with concentrations of 1000 μg/mL, 500 μg/mL, 250 μg/mL, 125 μg/mL, and 62.5 μg/mL. Exactly 10 μL of both reference substance and sample solutions were injected into the Agilent 1260 liquid chromatograph. The analyses were performed following predefined chromatographic conditions. The findings are outlined in S1 Table and illustrated in S2 Fig.

**S1 Table**. The standard curve results for geniposide, chlorogenic acid, quercetin, and 6-gingerol

| Reference Substance | Standard curve equation | *r*^2^ | Range of linearity |
| --- | --- | --- | --- |
| Geniposide | *y*=7.388*x*-3.8761 | 0.9999 | 205.0μg/mL~12.8μg/mL |
| Chlorogenic acid | *y*=38.646*x*+365.02 | 0.9999 | 255.0μg/mL ~ 15.94μg/mL |
| Quercetin | *y*=18.326*x*-11.899 | 0.9999 | 290.0μg/mL ~ 18.125μg/mL |
| 6-gingeror | *y=11.673x+51.003* | 1 | 1000μg/mL ~ 62.5μg/mL |

**S2 Table**. Sample results for geniposide, chlorogenic acid, quercetin, and 6-gingerol

| Reference Substance | Sample | 1 | 2 | 3 | Mean | SD | Unit |
| --- | --- | --- | --- | --- | --- | --- | --- |
| Chlorogenic acid | GF | 5.66 | 5.72 | 5.77 | 5.72 | 0.06 | mg/g |
|  | GFPG | 5.24 | 5.28 | 5.20 | 5.24 | 0.04 |  |
| Geniposide | GF | 113.37 | 113.72 | 113.51 | 113.53 | 0.18 | mg/g |
|  | GFPG | 98.09 | 97.85 | 97.26 | 97.73 | 0.43 |  |
| Quercetin | GF | 34.80 | 36.65 | 37.81 | 36.42 | 1.52 | ug/g |
|  | GFPG | 57.97 | 58.36 | 57.97 | 58.10 | 0.23 |  |
| 6-gingerol | GFPG | 1.50 | 1.51 | 1.51 | 1.51 | 0.01 | mg/g |

**S2 Fig**. HPLC chromatogram for geniposide, chlorogenic acid, quercetin, and 6-gingerol.

a-1, a-2, a-3 showed the chromatograms of geniposide in the reference substance, GF, and GFPG, respectively. b-1, b-2, b-3 showed the chromatograms of chlorogenic acid in the reference substance, GF, and GFPG, respectively. c-1, c-2, c-3 showed the chromatograms of quercetin in the reference substance, GF, and GFPG, respectively. d-1, d-2 showed the chromatograms of 6-gingerol in the reference substance and GFPG, respectively.
