# Supplementary material for: Optimized processing of Gardenia Fruits with ginger juice: Unveiling therapeutic mechanisms for cholestatic liver injury through TLR4/NF-κB, FXR/PPAR-α, and PI3K/AKT/GSK-3β
Source: PLoS One. 2025 Sep 16;20(9):e0330189. doi: 10.1371/journal.pone.0330189 (PMC12440179; doi:10.1371/journal.pone.0330189)
Supplement: S2 File — (PDF) [file pone.0330189.s007.pdf]

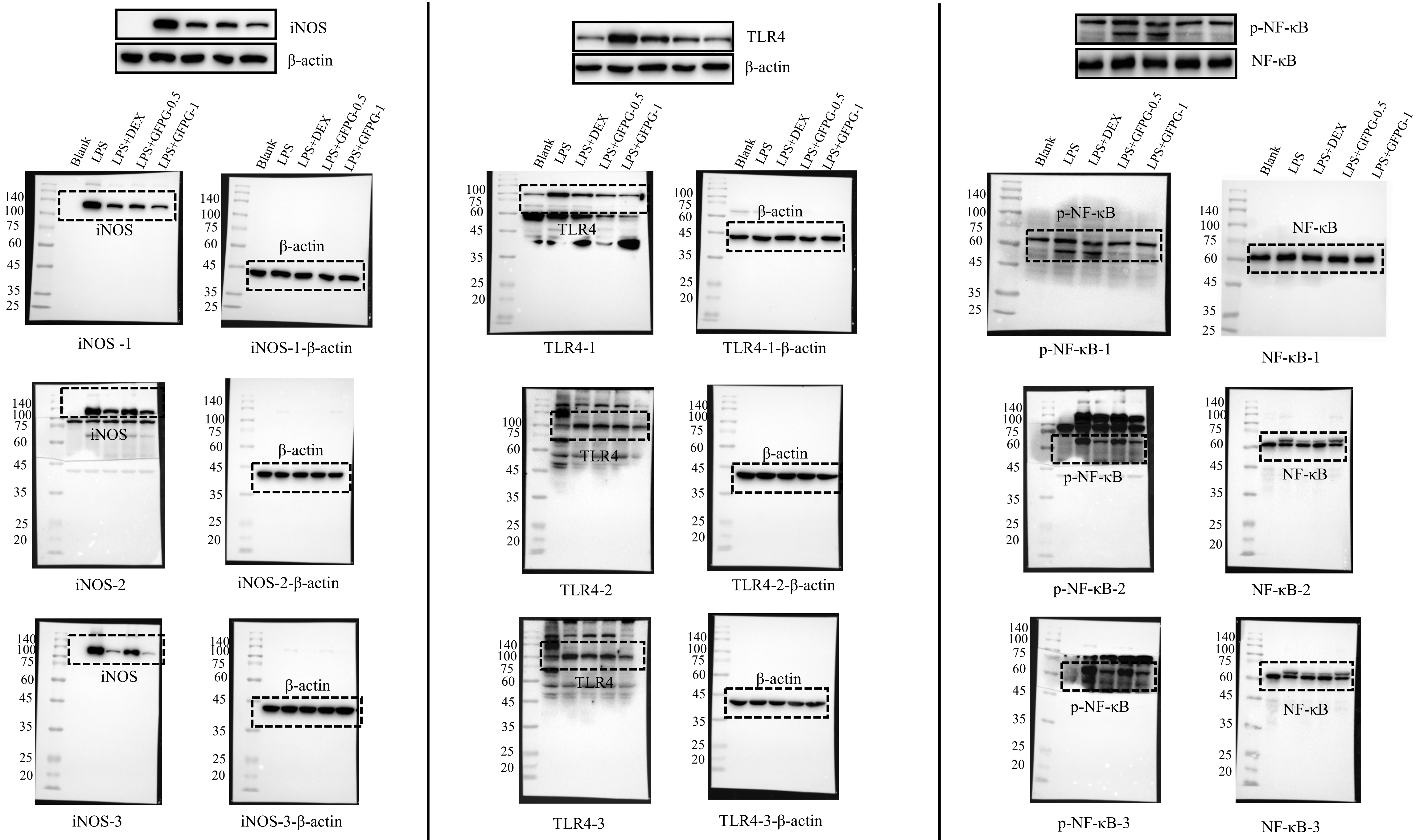
iNOS
β-actin
TLR4
β-actin
p-NF-κB
NF-κB
Blank
LPS
LPS+DEX
LPS+GFPG-0.5
LPS+GFPG-1
iNOS -1
iNOS-1-β-actin
iNOS-2
iNOS-2-β-actin
iNOS-3
iNOS-3-β-actin
TLR4-1
TLR4-1-β-actin
TLR4-2
TLR4-2-β-actin
TLR4-3
TLR4-3-β-actin
p-NF-κB-1
NF-κB-1
p-NF-κB-2
NF-κB-2
p-NF-κB-3
NF-κB-3

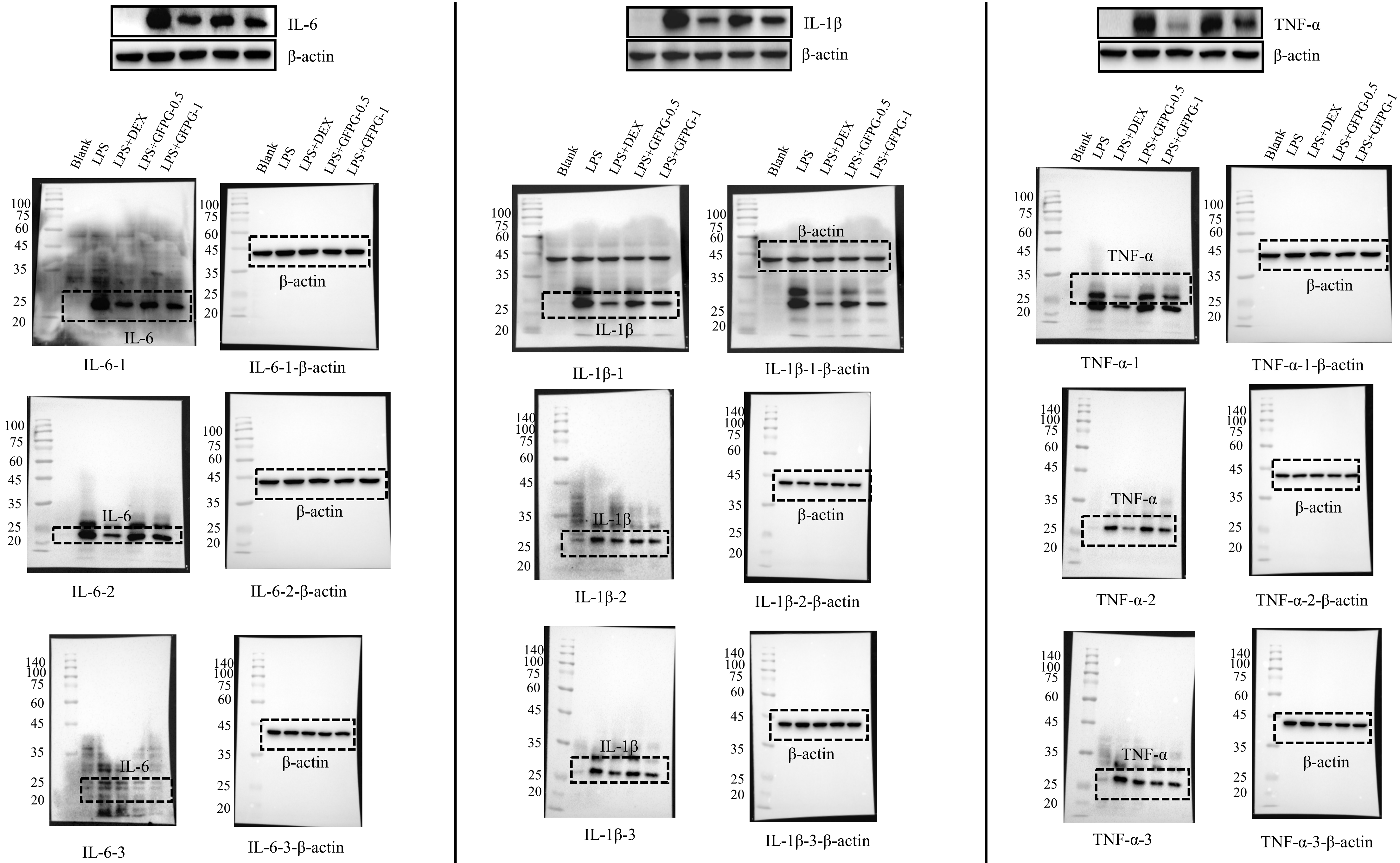
IL-6
β-actin
IL-1β
β-actin
TNF-α
β-actin
Blank
LPS
LPS+DEX
LPS+GFPG-0.5
LPS+GFPG-1
100
75
60
45
35
25
20
140
IL-6
β-actin
IL-1β
TNF-α
IL-6-1
IL-6-1-β-actin
IL-6-2
IL-6-2-β-actin
IL-6-3
IL-6-3-β-actin
IL-1β-1
IL-1β-1-β-actin
IL-1β-2
IL-1β-2-β-actin
IL-1β-3
IL-1β-3-β-actin
TNF-α-1
TNF-α-1-β-actin
TNF-α-2
TNF-α-2-β-actin
TNF-α-3
TNF-α-3-β-actin

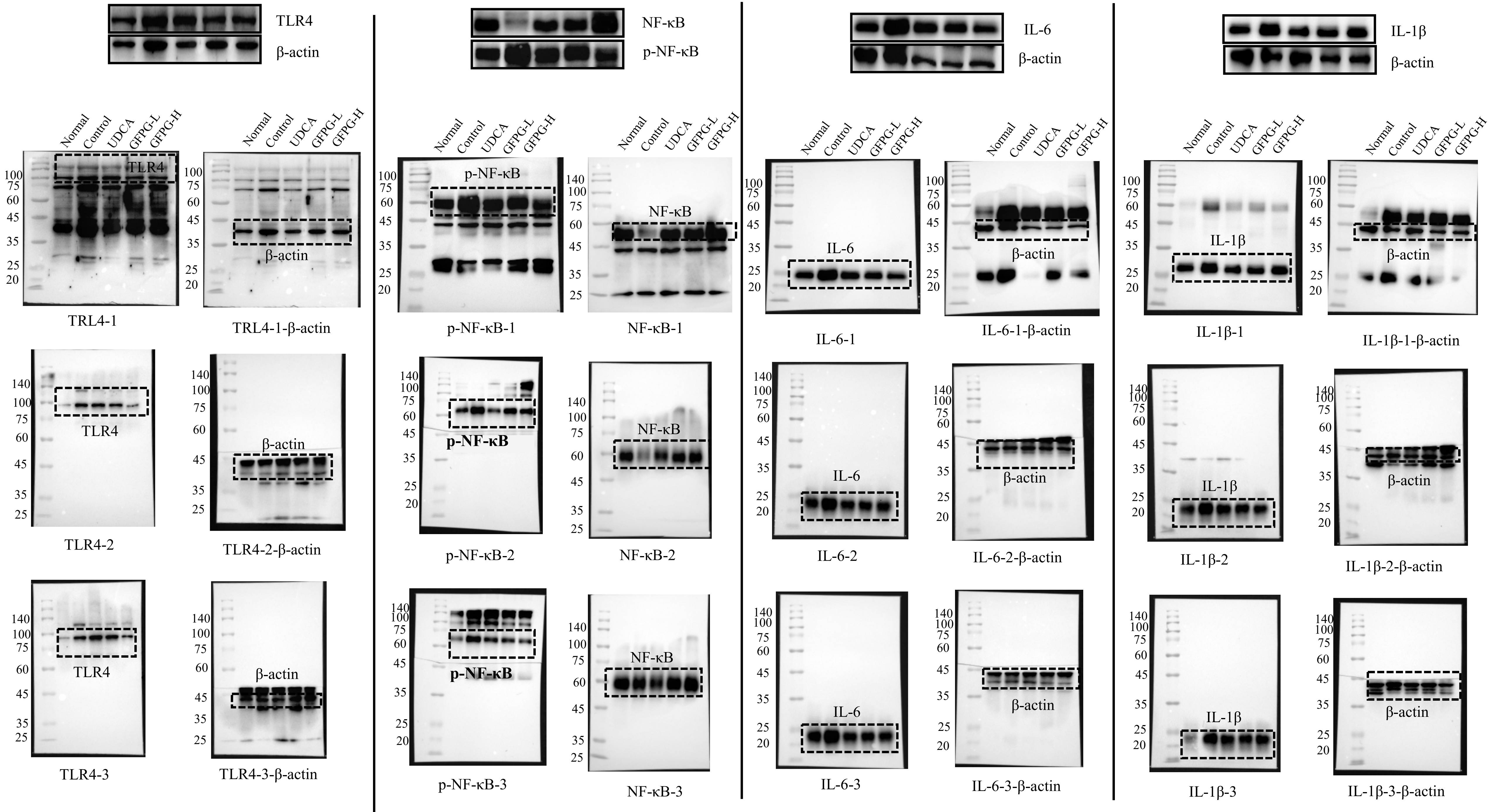
TLR4
β-actin
NF-κB
p-NF-κB
IL-6
β-actin
IL-1β
β-actin
Normal
Control
UDCA
GFPG-L
GFPG-H
TRL4-1
TRL4-1-β-actin
p-NF-κB-1
NF-κB-1
IL-6-1
IL-6-1-β-actin
IL-1β-1
IL-1β-1-β-actin
TLR4-2
TLR4-2-β-actin
p-NF-κB-2
NF-κB-2
IL-6-2
IL-6-2-β-actin
IL-1β-2
IL-1β-2-β-actin
TLR4-3
TLR4-3-β-actin
p-NF-κB-3
NF-κB-3
IL-6-3
IL-6-3-β-actin
IL-1β-3
IL-1β-3-β-actin
140
100
75
60
45
35
25
20

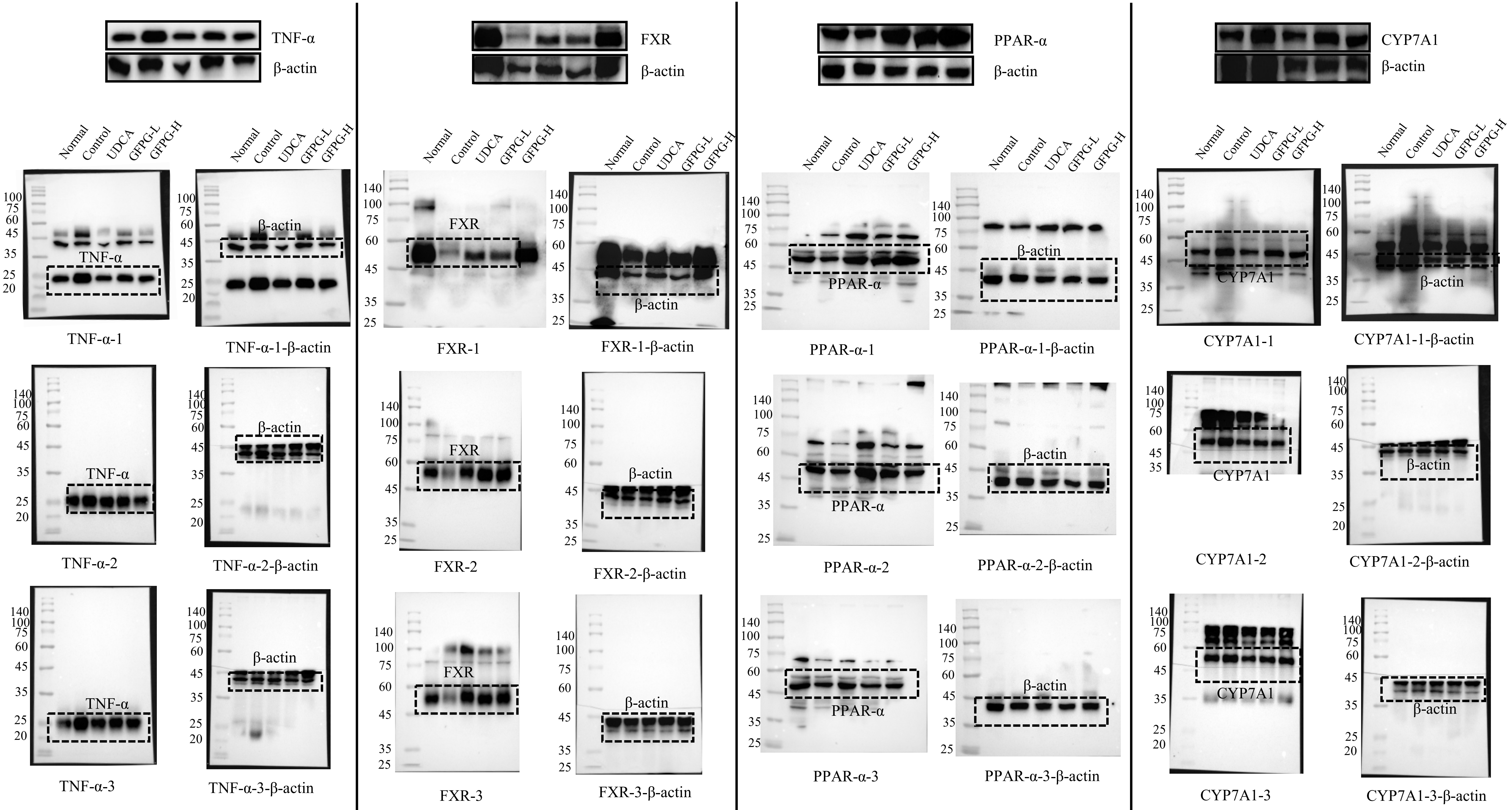
TNF-α
β-actin
FXR
β-actin
PPAR-α
β-actin
CYP7A1
β-actin
Normal
Control
UDCA
GFPG-L
GFPG-H
140
100
75
60
45
35
25
20
TNF-α-1
TNF-α-1-β-actin
TNF-α-2
TNF-α-2-β-actin
TNF-α-3
TNF-α-3-β-actin
FXR-1
FXR-1-β-actin
FXR-2
FXR-2-β-actin
FXR-3
FXR-3-β-actin
PPAR-α-1
PPAR-α-1-β-actin
PPAR-α-2
PPAR-α-2-β-actin
PPAR-α-3
PPAR-α-3-β-actin
CYP7A1-1
CYP7A1-1-β-actin
CYP7A1-2
CYP7A1-2-β-actin
CYP7A1-3
CYP7A1-3-β-actin

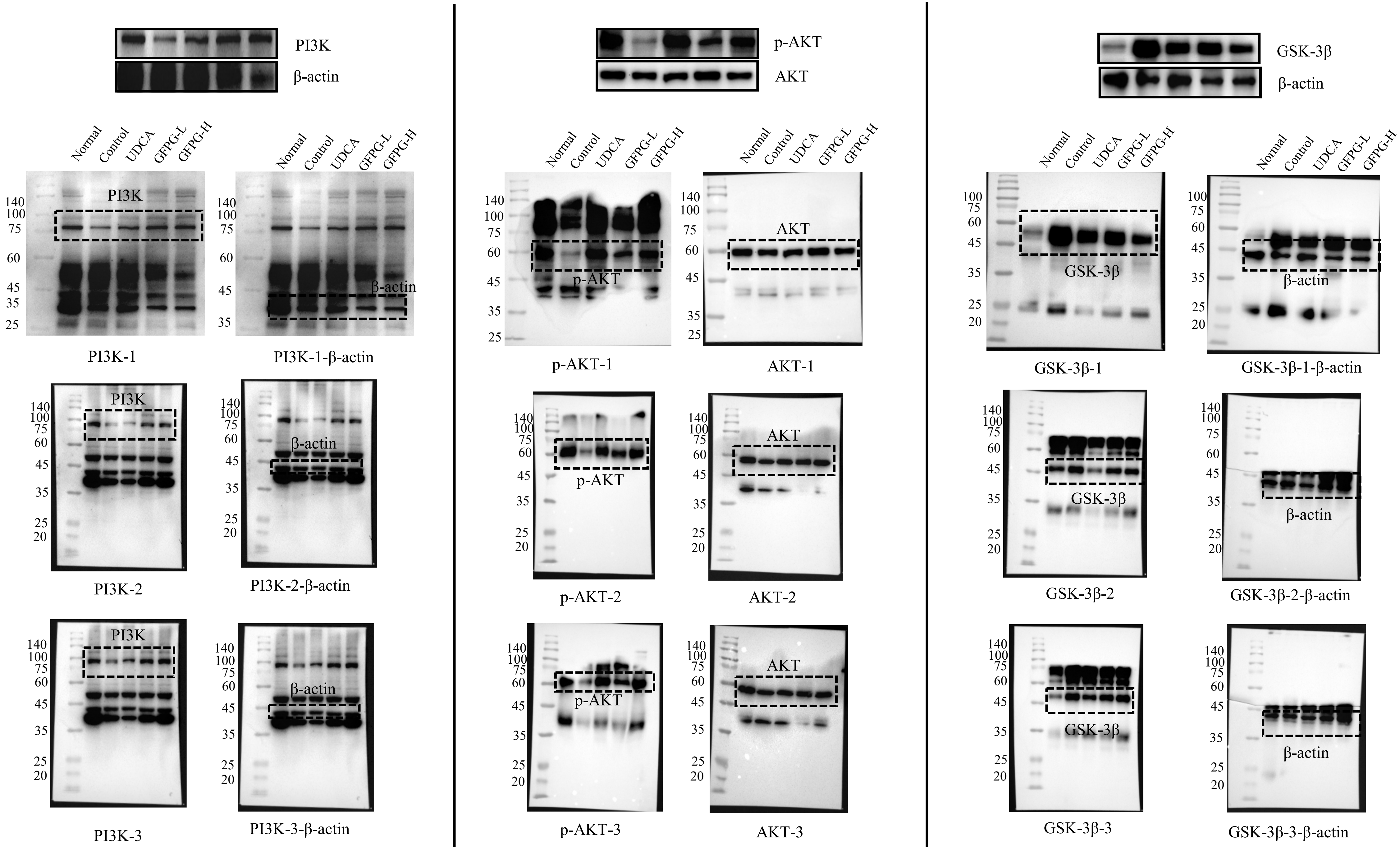
PI3K
β-actin
Normal
Control
UDCA
GFPG-L
GFPG-H
PI3K-1
PI3K-1-β-actin
PI3K-2
PI3K-2-β-actin
PI3K-3
PI3K-3-β-actin
p-AKT
AKT
p-AKT-1
AKT-1
p-AKT-2
AKT-2
p-AKT-3
AKT-3
GSK-3β
GSK-3β-1
GSK-3β-1-β-actin
GSK-3β-2
GSK-3β-2-β-actin
GSK-3β-3
GSK-3β-3-β-actin

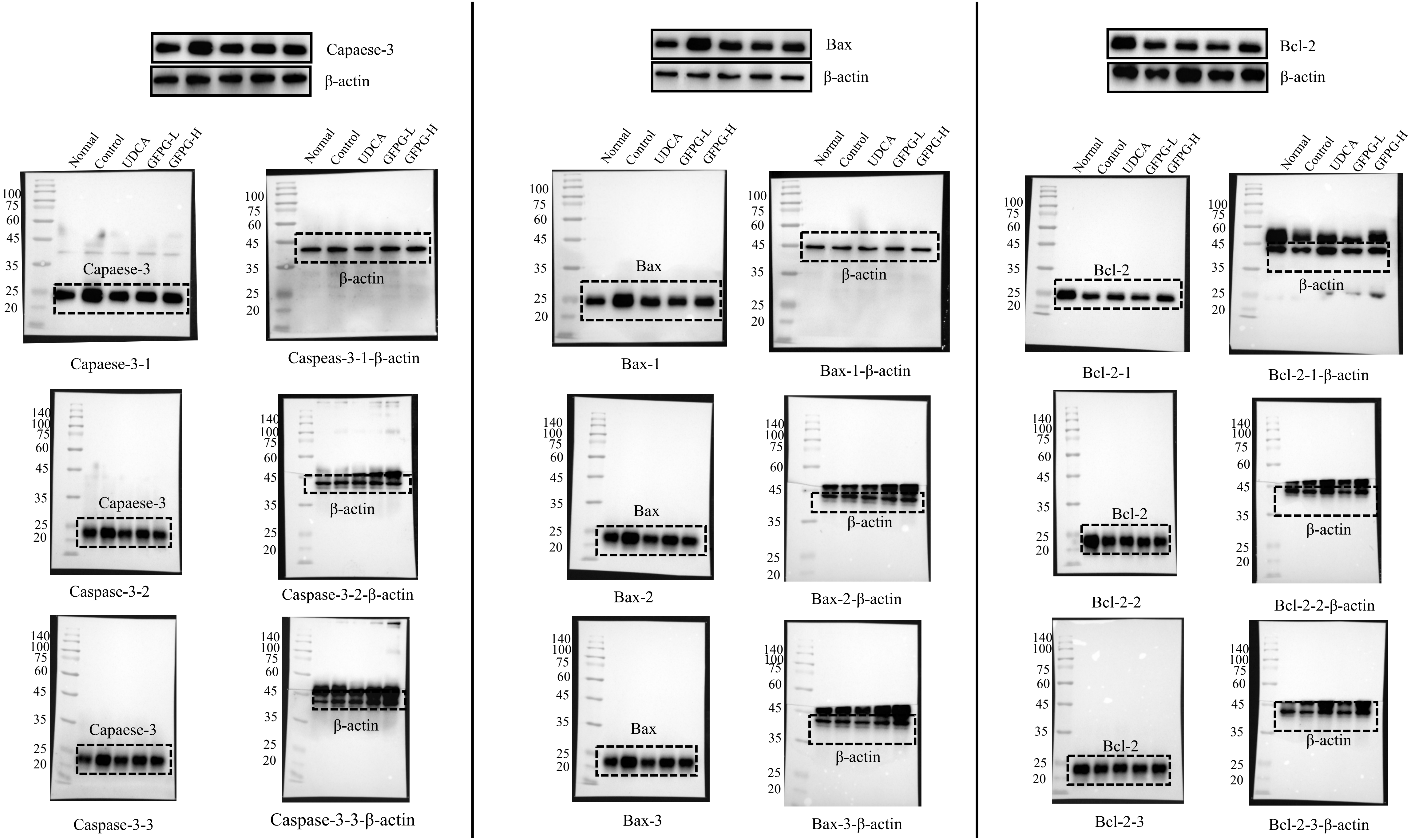
Capaese-3
β-actin
Bax
β-actin
Bcl-2
β-actin
Normal
Control
UDCA
GFPG-L
GFPG-H
140
100
75
60
45
35
25
20
Capaese-3
Capaese-3-1
β-actin
Caspeas-3-1-β-actin
Capaese-3
Caspase-3-2
β-actin
Caspase-3-2-β-actin
Capaese-3
Caspase-3-3
β-actin
Caspase-3-3-β-actin
Bax
Bax-1
β-actin
Bax-1-β-actin
Bax
Bax-2
β-actin
Bax-2-β-actin
Bax
Bax-3
β-actin
Bax-3-β-actin
Bcl-2
Bcl-2-1
β-actin
Bcl-2-1-β-actin
Bcl-2
Bcl-2-2
β-actin
Bcl-2-2-β-actin
Bcl-2
Bcl-2-3
β-actin
Bcl-2-3-β-actin
